# Characterization of Seed Endophytic Microbiota in *Pinus massoniana*

**DOI:** 10.3390/microorganisms14010199

**Published:** 2026-01-15

**Authors:** Yuhang Liu, Dongzhen Li, Yongxia Li, Xuan Wang, Wei Zhang, Xiaojian Wen, Zhenkai Liu, Yuqian Feng, Wandong Yin, Can Yang, Xingyao Zhang

**Affiliations:** 1 Key Laboratory of Forest Protection of National Forestry and Grassland Administration, Ecology and Nature Conservation Institute, Chinese Academy of Forestry, Beijing 100091, China; liuyuhang_cn@163.com (Y.L.); lidongzhen1949@163.com (D.L.); jiuwozhidao@163.com (X.W.); zhangwei1@caf.ac.cn (W.Z.); wenxj@caf.ac.cn (X.W.); zhenkailiu@caf.ac.cn (Z.L.); fengyuqian1988@163.com (Y.F.); wojiaowandong1@163.com (W.Y.); yangcan0428@163.com (C.Y.); zhangxingyao@126.com (X.Z.); 2 Co-Innovation Center for Sustainable Forestry in Southern China, Nanjing Forestry University, Nanjing 210073, China

**Keywords:** seed endophytic microbiota, *Pinus massoniana*, core seed microbiota, functional prediction

## Abstract

Seed endophytic microbiota are crucial for plant early development and stress resistance. *Pinus massoniana* is a key ecological and economic tree species in China, yet it is severely threatened by pine wilt disease (PWD). However, the community composition of *P. massoniana* seed endophytic microbiota and the persistent symbiosis formed via vertical transmission in seeds remain unclear. We analyzed the endophytic bacterial and fungal microbiota of *P. massoniana* seeds from four geographic regions using high-throughput 16S rRNA and ITS sequencing to characterize community structure, diversity, and functional potential, providing a basis for endophytic microbiota-based strategies to enhance resistance to PWD. Results showed that both alpha and beta diversity analyses indicated that seed endophytic microbial communities of *P. massoniana* differed among regions. Bacterial communities were dominated by Pseudomonadota (phylum), Gammaproteobacteria (class), and the genera *Klebsiella*, *norank_f_Pectobacteriaceae*, and *Lactobacillus*. Fungal communities were primarily composed of Ascomycota and Basidiomycota (phylum), Sordariomycetes (class), and the genera *Rosellinia*, *Aspergillus*, and *Coniophora*. Correlation network analysis revealed that fungal networks were characterized by a higher proportion of positive correlations, whereas bacterial networks were more complex. Notably, several genera detected in seeds, including *Pseudomonas*, *Bacillus*, and *Trichoderma*, have also been reported in mature *P. massoniana* tissues, indicating a potential for putative vertical transmission from mother plants. Functional prediction further suggested that these taxa were enriched in pathways related to terpenoid and polyketide metabolism and saprotrophic functions, which have been implicated in PWD resistance and have been previously reported to exert nematode-suppressive or plant growth-promoting effects. Overall, this study elucidates the community structure and ecological characteristics of seed endophytic microbiota in *P. massoniana* and identifies potentially beneficial microbial taxa, providing potential support for the future utilization of *P. massoniana* endophytic microbiota in PWD research.

## 1. Introduction

Seeds, as pivotal carriers of genetic information and reproductive organs of plants, have evolved sophisticated adaptive mechanisms to navigate environmental stresses over evolutionary timescales [1,2]. Central to these mechanisms are seed endophytic microbiota, which colonize internal tissues and establish intimate associations with hosts during early developmental stages, such as germination and seedling establishment [3,4]. These microbial symbionts contribute to host fitness through multifaceted pathways, including direct facilitation of nutrient acquisition (e.g., nitrogen fixation, phosphate solubilization) and phytohormone biosynthesis [2,5,6], as well as indirect enhancement of stress resilience via induction of systemic resistance and production of antimicrobial secondary metabolites, thereby emerging as a focal frontier in plant endophytic microbiota research.

Recent breakthroughs in high-throughput sequencing technologies have significantly advanced our understanding of seed endophytic microbiota in crop species, such as wheat and rice, where they have been linked to improved germination rates and stress tolerance [7,8,9]. However, comparable research in forest tree species—particularly ecologically and economically vital conifers—remains disproportionately underdeveloped. Existing studies have largely been restricted to a few species. For example, in *Pinus monticola*, seeds were found to harbor distinct fungal communities compared with needles, with dominant foliar taxa such as *Lophodermium* spp. absent from seeds [10]. Similarly, cultivable bacteria, including strains with antagonistic activity against phytopathogens, have been isolated from *Picea abies* seeds [11]. Methylotrophic and methanotrophic bacteria have also been detected in seeds and other tissues of *Pinus sylvestris* and *Picea pungens*, indicating persistent associations with woody plants and potential roles in growth promotion and stress tolerance [12]. The community structure, core taxonomic constituents, and functional repertoire of seed endophytic microbiota in coniferous trees remain largely uncharacterized, limiting insights into their roles in shaping host health and adaptive strategies against biotic and abiotic challenges.

*P. massoniana*, a keystone native conifer in subtropical China, plays an indispensable role in soil conservation, carbon sequestration, and timber production. Yet, its populations face escalating threats from pine wilt disease (PWD), a devastating affliction triggered by infection with the pine wood nematode (PWN, *Bursaphelenchus xylophilus*) and disseminated by *Monochamus* spp. beetles. PWD has inflicted profound ecological and economic impacts, triggering widespread tree mortality and disrupting forest ecosystem stability [13]. Previous studies on *P. massoniana*-related microbiota have mainly focused on rhizosphere soils and woody tissues under different environmental or physiological contexts [14,15]. Emerging evidence underscores the critical role of early-life endophytic microbiota assembly in determining plant health trajectories [16], with seeds serving as the foundational niche for initial microbial colonization. Seed-transmitted microbiota may undergo vertical inheritance [17,18], establishing persistent symbioses that influence host resistance to PWD across developmental stages.

Despite the recognized potential of seed endophytic microbiota as mediators of host resilience, the compositional architecture, geographic variability, and functional traits of *P. massoniana* seed endophytic microbiota remain largely elusive. Elucidating these aspects is essential for deciphering the dynamics of plant-microbe interactions and developing innovative, endophytic microbiota-centric strategies for PWD management. Accordingly, this study sought to profile the composition and organization of endophytic bacterial and fungal communities within *P. massoniana* seeds across four distinct geographic regions, identify core endophytic microbial taxa and their functional profiles, and explore potential links between seed endophytic microbiota composition, geographic variation, and host resistance to PWD.

## 2. Materials and Methods

### 2.1. Study Area and Sampling

Mature cones of *P*. *massoniana* were collected from four representative regions in China: Guigang, Guangxi Province (GX; 23°11′ N, 109°48′ E); Jurong, Jiangsu Province (JS; 32°12′ N, 119°24′ E); Fenyi, Jiangxi Province (JX; 27°53′ N, 114°54′ E); and Junan, Shandong Province (SD; 35°16′ N, 118°59′ E). At each site, fifteen healthy, disease-free adult *P. massoniana* mother trees (≥15 years old), with a minimum distance of 30 m between any two trees to reduce potential confounding effects of genetic relatedness and spatial proximity. For each tree, five mature and closed cones were collected from the upper to middle canopy using pole pruners or climbing equipment, while avoiding ground contact. In total, 300 cones were collected across the four regions. Cones from each tree were individually placed in sterile sealed bags, labeled, and kept under refrigerated conditions (4 °C) during transport to the laboratory. All samples were processed within 48 h after collection.

In the laboratory, cone scales were removed under sterile conditions, and the extracted seeds were cleaned of visible debris and air-dried for 24 h to remove any remaining surface moisture (Figure S1). Subsequently, seed coats were manually removed, and the seeds were surface sterilized by stepwise treatment with 70% ethanol containing 0.1% Triton X-100 (5 min), 2.5% sodium hypochlorite (5 min), and 70% ethanol (30 s), followed by five rinses with sterile distilled water [19,20]. This procedure was performed to eliminate epiphytic microorganisms and thereby ensure that only seed-borne endophytic microorganisms were analyzed. The final rinse water was plated on potato dextrose agar (PDA, BD, Franklin Lakes, NJ, USA) and nutrient agar (NA, Beijing Aoboxing Bio-Tech Co., Ltd., Beijing, China) media to verify the effectiveness of surface sterilization. Seeds from each tree were pooled and transferred into sterile 2 mL microcentrifuge tubes. Each tube contained approximately 150 dehulled and surface-sterilized seeds (approximately 1.0 g per tube), resulting in a total of fifteen tubes per region. In total, 60 seed samples were obtained. All tubes were rapidly frozen in liquid nitrogen and kept at −80 °C for no more than two weeks before DNA extraction to preserve the integrity of the microbial communities.

### 2.2. DNA Extraction and PCR Amplification

Microbial genomic DNA was isolated from seed samples using the FastDNA^®^ Spin Kit for Soil (MP Biomedicals, Irvine, CA, USA) following the manufacturer’s protocol [21]. DNA quality and yield were assessed by electrophoresis on a 1.0% agarose gel and quantified using a NanoDrop 2000 spectrophotometer (Thermo Scientific, Waltham, MA, USA). Purified DNA samples were stored at −80 °C until downstream analyses. PCR amplification was carried out on an ABI GeneAmp^®^ 9700 thermal cycler (Applied Biosystems, Carlsbad, CA, USA) using seed-derived microbial DNA as the template. Bacterial 16S rRNA gene fragments were amplified with primers 799F (5′-AACMGGATTAGATACCCKG-3′) and 1193R (5′-ACGTCATCCCCACCTTCC-3′), while fungal ITS regions were amplified using primers ITS1F (5′-CTTGGTCATTTAGAGGAAGTAA-3′) and ITS2R (5′-GCTGCGTTCTTCATCGATGC-3′) [22,23]. Each 20 μL PCR reaction contained 10 μL of FastPfu Polymerase (TransGen Biotech Co., Ltd., Beijing, China), 0.8 μL of each primer (5 μM), approximately 10 ng of template DNA, and nuclease-free water. The thermal cycling program consisted of an initial denaturation at 95 °C for 3 min, followed by 13 cycles for bacterial amplification or 35 cycles for fungal amplification at 95 °C for 30 s, 55 °C for 30 s, and 72 °C for 45 s, with a final extension at 72 °C for 10 min. After DNA quality assessment and sequencing library quality control, samples that did not meet the quality criteria were excluded. Ultimately, 36 qualified samples were retained for downstream sequencing and analyses, including 10 samples from GX, 8 from JS, 9 from JX, and 9 from SD.

Amplified products were visualized on a 2% agarose gel, excised, and purified using a PCR Clean-Up Kit (YuHua, Shanghai, China) in accordance with the manufacturer’s instructions. Purified amplicons were quantified with a Qubit 4.0 fluorometer (Thermo Fisher Scientific, Waltham, MA, USA).

### 2.3. Illumina Sequencing

Library preparation of the purified PCR products was performed using the NEXTFLEX Rapid DNA-Seq Kit (PerkinElmer, Waltham, MA, USA). All amplicon libraries were uniquely barcoded to distinguish samples and replicates, and sequencing was performed as 2 × 150 bp paired-end reads on an Illumina NextSeq 2000 platform (Illumina, San Diego, CA, USA) by Majorbio Bio-Pharm Technology Co. Ltd. (Shanghai, China) in accordance with their established workflows [24]. Prior to sequencing, purified amplicons were normalized and combined at equimolar concentrations.

### 2.4. Data Processing

Raw FASTQ reads were first de-multiplexed using a custom Perl script. Quality filtering was performed with fastp (v0.19.6) [25] and paired-end reads were merged using FLASH (v1.2.7) [26] under the following conditions: reads were truncated if the average quality score within a 50 bp sliding window fell below 20, and truncated reads shorter than 50 bp were discarded. Reads containing ambiguous bases were removed, and only overlapping sequences exceeding 10 bp were merged, allowing a maximum mismatch ratio of 0.2 in the overlap region. Unassembled reads were discarded. Sequences were then assigned to samples based on barcodes and primers, permitting exact barcode matches and up to two mismatches in primer sequences, and the orientation of sequences was corrected accordingly. Optimized sequences were clustered into operational taxonomic units (OTUs) at 97% similarity using UPARSE v7.1 [27,28], a threshold widely adopted to balance taxonomic resolution and computational efficiency while minimizing sequence inflation due to errors. For each OTU, the most abundant sequence was selected as its representative. Chloroplast-derived sequences were removed from all samples to avoid plant DNA interference. To control for differences in sequencing depth, the number of sequences per sample was rarefied to the minimum sequence count observed, resulting in an average Good’s coverage of 99.99%. Alpha diversity metrics, including observed OTUs, Chao1, and ACE, were calculated to assess microbial richness and evenness. Chao1 estimates total OTU richness, with higher values reflecting greater diversity, whereas ACE incorporates both richness and evenness, providing a measure of community uniformity.

Taxonomic assignment of OTU representative sequences was conducted using RDP Classifier v2.2 [29] against the SILVA 138 database for 16S rRNA and UNITE 8.0 database for ITS sequences, applying the recommended confidence threshold of 0.7 to ensure reliable classification while maintaining ecological variability. Functional predictions for bacterial communities were performed with PICRUSt2 v2.2.0 [30], whereas fungal ecological roles were inferred using FUNGuild v1 [31].

### 2.5. Statistical Analysis

Bioinformatic analyses were performed on the Majorbio Cloud platform (https://cloud.majorbio.com (accessed on 20 January 2025) [32]. Using the OTU dataset, rarefaction curves and alpha diversity metrics were calculated with Mothur v1.30.1 [33]. Bray–Curtis distance matrices were calculated to assess the beta diversity of bacterial and fungal endophytic communities in *P*. *massoniana* seeds. Principal Coordinate Analysis (PCoA) ordinations were used to visualize community differences. Differences in community composition among regions were tested using one-way PERMANOVA and ANOSIM with 999 permutations. Homogeneity of multivariate dispersion was evaluated using PERMDISP/betadisper with 999 permutations. Venn diagrams were generated using OTU tables grouped at the genus level, with OTUs assigned to genera based on taxonomy. The number of shared and unique genera among different sample groups was calculated, and the analysis and visualization were carried out in R v4.5.2. Co-occurrence networks were constructed to investigate the relationships within microbial communities across samples [34]. Microbial correlation matrices were generated in R using the “psych” package [35]. A correlation between two nodes was considered to represent a potential interaction when Spearman’s ρ > 0.8 or <−0.8 with *p* ≤ 0.05. Correlation networks were visualized using Gephi version v0.10.1. Network modularity was calculated with a resolution parameter of 1.0, and genus-level within-module (Zi) and among-module (Pi) connectivity values were determined according to the established framework [36].

## 3. Results

### 3.1. Diversity of Seed Endophytic Microbial Communities in P. massoniana

#### 3.1.1. Bacterial Community Diversity in *P. massoniana* Seeds

**Figure 1:**
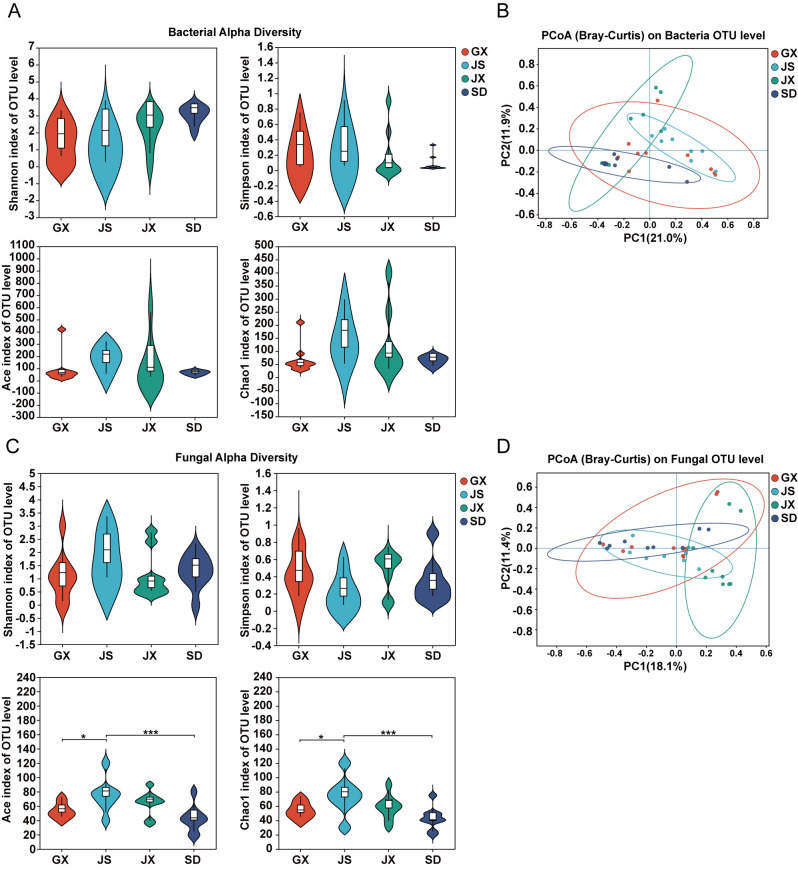
Diversity of seed endophytic microbial communities in *P. massoniana*. (**A**) Alpha diversity of seed endophytic bacterial communities, including Shannon, Simpson, ACE, and Chao1 indices. (**B**) Principal coordinates analysis (PCoA) of bacterial communities based on Bray–Curtis dissimilarities calculated from the OTU table; ellipses denote 95% confidence regions. Group differences in community composition were assessed using one-way PERMANOVA and ANOSIM, and homogeneity of multivariate dispersion was evaluated using PERMDISP/betadisper. (**C**,**D**) Corresponding alpha diversity indices (**C**) and beta diversity (**D**) of seed endophytic fungal communities. One-way ANOVA followed by Tukey’s post hoc test was used to evaluate significant differences. “*” indicate *p* ≤ 0.05. “***” indicated *p* ≤ 0.001.

A total of 4,536,699 high-quality bacterial 16S rRNA reads were obtained from 36 samples, of which 4,365,754 reads (quality score ≥ 20) were clustered into 1095 OTUs. Taxonomic annotation classified these sequences into 1 domain, 1 kingdom, 29 phyla, 74 classes, 190 orders, 331 families, 597 genera, and 841 species. Alpha diversity analysis showed no significant differences in bacterial community diversity (Simpson, Shannon, ACE and Chao1 indices) among *P. massoniana* seeds from the four regions (Figure 1A). PCoA based on Bray–Curtis dissimilarities of the OTU table revealed a clear geographic structuring of the endophytic bacteria communities in *P. massoniana* seeds from four provinces in China. The first two axes explained 21.0% (PC1) and 11.9% (PC2) of the total variation (32.9% cumulatively), and samples tended to cluster by province with only partial overlap among groups. Notably, JX samples were shifted toward higher PC2 values, whereas JS samples were generally displaced along the positive PC1 direction; SD samples were concentrated toward the negative region of the ordination space, and GX samples exhibited a comparatively broader spread while remaining distinct in centroid position relative to other provinces. Permutation-based tests supported these patterns: one-way PERMANOVA indicated significant differences in community composition among provinces (F = 2.440, *p* = 0.001; 999 permutations), consistent with ANOSIM (R = 0.300, *p* = 0.001; 999 permutations). The homogeneity of multivariate dispersion was not significant (PERMDISP/betadisper: F = 2.884, *p* = 0.079; 999 permutations), suggesting that the detected PERMANOVA signal is primarily attributable to shifts in community composition among provinces rather than differences in within-group dispersion (Figure 1B).

#### 3.1.2. Fungal Community Diversity in *P. massoniana* Seeds

A total of 5,397,382 high-quality fungal ITS reads were obtained from 36 samples, of which 5,174,460 reads (quality score ≥ 20) were clustered into 719 OTUs. Taxonomic annotation classified these sequences into 1 domain, 1 kingdom, 8 phyla, 29 classes, 74 orders, 167 families, 293 genera, and 405 species. Alpha diversity analysis revealed no significant differences in fungal community diversity, as measured by the Simpson and Shannon indices, among *P. massoniana* seed samples from different regions. However, the ACE and Chao1 indices showed significant differences. Specifically, the JS region exhibited the highest alpha diversity, with ACE and Chao1 values significantly higher than those of GX (*p* ≤ 0.05) and even more significantly higher than those of SD (*p* ≤ 0.001), but not significantly different from those of JX (Figure 1C). PCoA based on Bray–Curtis dissimilarities of the fungal OTU table revealed a pronounced geographic structuring of the endophytic fungal communities in *P. massoniana* seeds across four provinces. The first two axes explained 18.1% (PC1) and 11.4% (PC2) of the total variation (29.5% cumulatively). Samples tended to cluster by province in the ordination space, with 95% confidence ellipses indicating group-level separation and only partial overlap among provinces. Permutation-based tests corroborated these patterns: one-way PERMANOVA detected significant differences in community composition among provinces (F = 2.5309, *p* = 0.001; 999 permutations), consistent with ANOSIM (R = 0.3569, *p* = 0.001; 999 permutations). Importantly, the homogeneity of multivariate dispersion did not differ significantly among provinces (PERMDISP/betadisper: F = 1.8729, *p* = 0.139; 999 permutations), indicating that the observed PERMANOVA signal is primarily driven by shifts in community composition rather than unequal within-group dispersion (Figure 1D).

### 3.2. Composition of Seed Endophytic Microbial Communities

#### 3.2.1. Taxonomic Composition of Bacterial Communities

**Figure 2:**
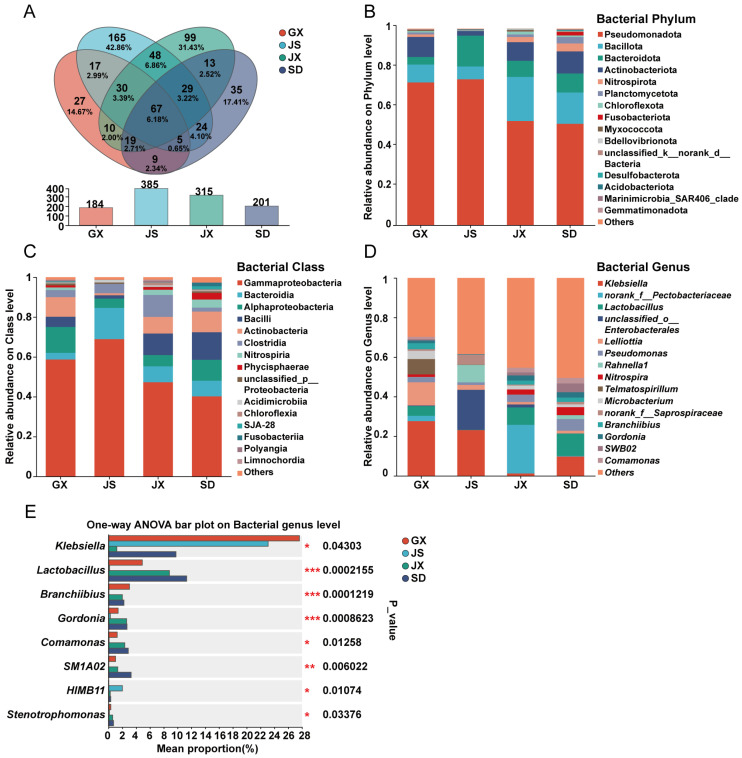
Taxonomic composition of seed endophytic bacterial communities in *P. massoniana*. (**A**) Venn diagram showing unique and shared bacterial genera among regions. (**B**–**D**) Relative abundances of seed endophytic bacterial taxa at the phylum (**B**), class (**C**), and genus (**D**) levels. Only the top 15 most abundant taxa are shown. (**E**) Differential abundance analysis of bacterial genera across regions. The rightmost column indicated *p*-values, with asterisks denoting significance levels. One-way ANOVA followed by Tukey’s post hoc test was used to evaluate significant differences. “*” indicated *p* ≤ 0.05. “**” indicated *p* ≤ 0.01. “***” indicated *p* ≤ 0.001.

Among the 597 identified bacterial genera, 67 were shared among all four regional groups (Figure 2A). The dominant bacterial phyla across all samples were Pseudomonadota, Bacillota, Bacteroidota, and Actinobacteriota (Figure 2B), with major classes including Gammaproteobacteria, Bacteroidia, Alphaproteobacteria, Bacilli, and Actinobacteria (Figure 2C). Among the universally shared genera, the top 10 in relative abundance were *Klebsiella*, *norank_f_Pectobacteriaceae*, *Lactobacillus*, *unclassified_o_Enterobacterales*, *Lelliottia*, *Pseudomonas*, *Rahnella1*, *Microbacterium*, *Branchiibius*, and *Gordonia* (Figure 2D). However, the abundance of these genera varied across regions. For example, *norank_f__Pectobacteriaceae* accounted for 24.55% in JX but only 2.69%, 0.02%, and 0.28% in GX, JS, and SD, respectively. *unclassified_o_Enterobacterales* was highly abundant in JS (20.20%), while *Lelliottia* was notably enriched in GX (11.72%). Significance testing of genus-level differences showed that eight genera—*Klebsiella*, *Lactobacillus*, *Branchiibius*, *Gordonia*, *Comamonas*, *SM1A02*, *HIMB11*, and *Stenotrophomonas*—exhibited significant or highly significant differences across the four regions. Specifically, *Lactobacillus*, *Branchiibius*, and *Gordonia* showed highly significant differences (*p* ≤ 0.001), *SM1A02* showed very significant differences (*p* ≤ 0.01), and the remaining four genera had significant differences (*p* ≤ 0.05) (Figure 2E). Detailed analysis revealed that *Lactobacillus*, *Branchiibius*, and *Gordonia* were present at extremely low abundances in seeds from JS, accounting for only 0.15%, 0.05%, and 0.26%, respectively—each less than 1% of the total bacterial community. In contrast, these three genera showed notably higher relative abundances in GX, JX, and SD: *Lactobacillus* was 4.89%, 8.82%, and 11.31%; *Branchiibius* was 3.03%, 2.00%, and 2.23%; and *Gordonia* was 1.39%, 2.61%, and 2.67%, respectively. The genus *SM1A02* was absent in JS samples but accounted for 1.02%, 1.33%, and 3.27% in GX, JX, and SD, respectively. Moreover, the relative abundance of *Klebsiella* was significantly lower in JX (1.21%) compared to GX (27.62%), JS (23.10%), and SD (9.76%). Both *Comamonas* and *Stenotrophomonas* also showed low abundance in JS, whereas *HIMB11* was most abundant in JS samples.

The Venn diagram (Figure 2A) was generated using genus-level OTU tables to assess shared and unique bacterial genera among regions. The results indicated that 27, 165, 99, and 35 genera were unique to GX, JS, JX, and SD, respectively. The top five unique genera for each region were as follows: GX: *Duganella* (33.21%), *Fusicatenibacter* (18.72%), *Asticcacaulis* (10.64%), *unclassified_f_Rhodocyclaceae* (7.71%), and *Dorea* (7.34%). JS: *Acholeplasma* (17.47%), *Maricaulis* (4.76%), *Anaerococcus* (3.72%), *Salinisphaera* (3.72%), and *norank_f_Erwiniaceae* (3.03%). JX: *norank_f_norank_o_Microtrichales* (12.57%), *Hyphomicrobium* (10.10%), *unclassified_o_Bacteroidales* (6.47%), *norank_f__SC-I-8* (6.41%), and *norank_f_TRA3-20* (6.35%). SD: *Ellin6067* (18.00%), *norank_f_KD3-93* (8.30%), *Haliscomenobacter* (8.07%), *Cellulomonas* (7.60%), and *Pelistega* (6.95%) (Table S1). Among pairwise comparisons, JS and JX shared the largest number of bacterial genera (174), with 48 being uniquely shared by these two regions, including *unclassified_o_Enterobacterales*, *NS5_marine_group*, *Vibrio*, and *Clostridium_sensu_stricto_12*, among others. In summary, although JS shared more genera with other regions, this was largely attributable to its higher overall bacterial richness and diversity. Overall, the bacterial communities in JS samples exhibited a distinct compositional pattern compared with those in the other three regions.

#### 3.2.2. Taxonomic Composition of Fungal Communities

**Figure 3:**
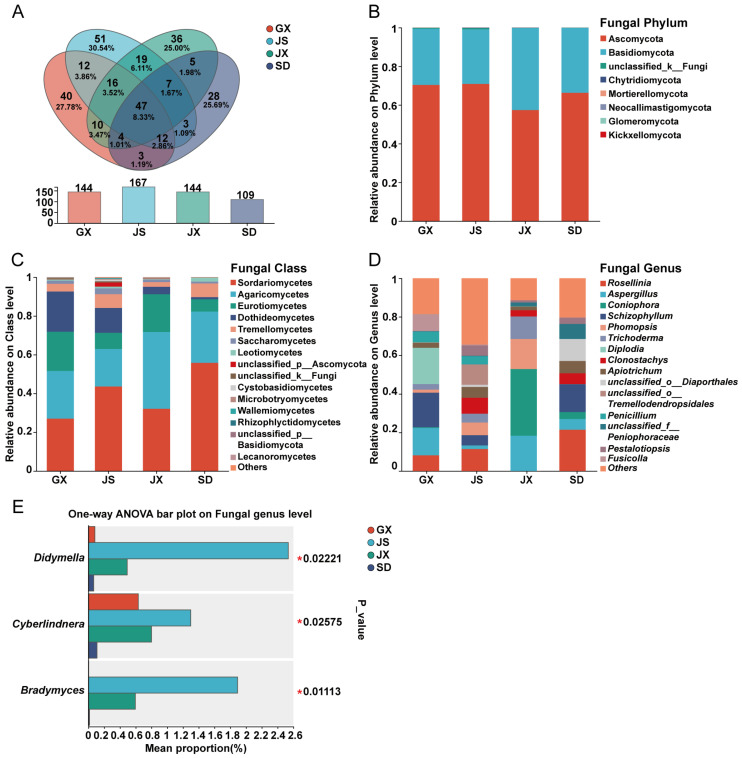
Taxonomic composition of seed endophytic fungal communities in *P. massoniana*. (**A**) Venn diagram showing unique and shared fungal genera among regions. (**B**–**D**) Relative abundances of seed endophytic fungal taxa at the phylum (**B**), class (**C**), and genus (**D**) levels. Only the top 15 most abundant taxa are shown. (**E**) Differential abundance analysis of fungal genera across regions. The rightmost column indicated *p*-values, with asterisks denoting significance levels. One-way ANOVA followed by Tukey’s post hoc test was used to evaluate significant differences. “*” indicated *p* ≤ 0.05.

Among the 293 identified fungal genera, 47 were shared across all four regions (Figure 3A). Fungal communities were dominated by two phyla—Ascomycota and Basidiomycota—which accounted for nearly all sequences in all samples (Figure 3B). The dominant classes included Sordariomycetes, Agaricomycetes, Eurotiomycetes, Dothideomycetes, and Tremellomycetes (Figure 3C). Among the genera shared by all regions, the top 10 in relative abundance were *Aspergillus*, *Schizophyllum*, *Phomopsis*, *Apiotrichum*, *unclassified_o_Diaporthales*, *Penicillium*, *unclassified_f_Peniophoraceae*, *Pestalotiopsis*, *Sistotrema*, and *Diaporthe* (Figure 3D). However, the relative abundances of these genera varied across regions. For example, *Aspergillus* was abundant in GX (14.26%) and JX (18.33%) but at much lower relative abundances in the other regions. *Schizophyllum* and *Phomopsis* were present at very low relative abundances in JX and SD, respectively. In contrast, *unclassified_o_Diaporthales* was significantly enriched in SD (11.41%) compared to the other three regions. Differential abundance testing at the genus level showed that *Didymella*, *Cyberlindnera*, and *Bradymyces* exhibited significant differences among the four regions (*p* ≤ 0.05) (Figure 3E). Specifically, *Didymella* was only detected in JS (2.54%) and JX (0.49%). *Cyberlindnera* showed similar abundance in GX (0.63%), JX (1.30%), and JS (0.80%), but was absent in SD. *Bradymyces* was detected only in JS (1.89%) and JX (0.59%), and was absent in GX and SD.

The Venn diagram (Figure 3A) was generated based on genus-level OTU tables to assess the numbers of shared and unique fungal genera among regions. It indicated that 40, 51, 36, and 28 fungal genera were unique to GX, JS, JX, and SD, respectively. The top five unique genera in GX were *Anthostomella* (25.48%), *unclassified_f_Hyaloscyphaceae* (20.38%), *Phialemonium* (9.81%), *Itersonilia* (9.67%), and *unclassified_o_Agaricales* (8.23%). In JS, only one genus (*unclassified_o_Tremellodendropsidales*) exceeded 1% in relative abundance, accounting for a dominant 87.48% of its unique genera. The top five unique genera in JX were *Scytalidium* (25.73%), *Spegazzinia* (10.02%), *unclassified_c_Lecanoromycetes* (8.20%), *Strelitziana* (8.15%), and *unclassified_o_Auriculariales* (6.30%). In SD, the top five unique genera included *Thelonectria* (44.43%), *Neopestalotiopsis* (37.25%), *Xylaria* (10.50%), *Pezicula* (2.67%), and *Cladophialophora* (1.95%) (Table S2). Consistent with the bacterial community patterns, JS and JX shared the greatest number of fungal genera (89), of which 19 were exclusively shared by the two regions, including *Didymella*, *Paraphoma*, and *Bradymyces*. In summary, JS and JX had more shared fungal genera and no significant differences in overall fungal diversity or richness. However, in terms of compositional structure, the fungal communities of JS and SD exhibited greater similarity in genus-level abundance profiles.

### 3.3. Co-Occurrence and Correlation Patterns of Seed Endophytic Microbial Communities

#### 3.3.1. Bacterial Co-Occurrence and Correlation Network

**Figure 4:**
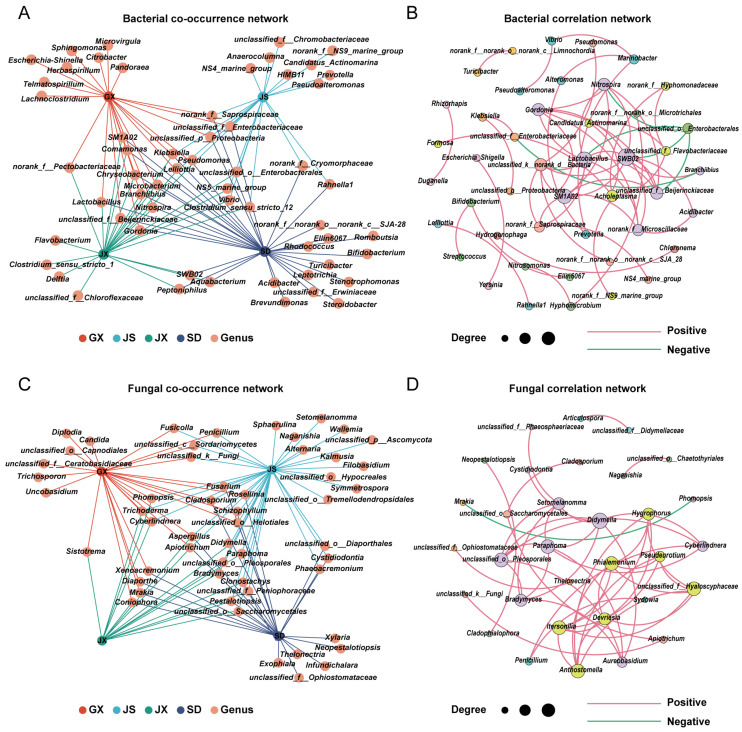
Co-occurrence and correlation networks of seed endophytic microbial genera in *P. massoniana*. (**A**) Co-occurrence network of seed endophytic bacterial genera across regions. (**B**) Correlation network of seed endophytic bacterial genera based on the top 100 most abundant genera. Node size represents the degree, node color indicates module membership, and edge color denotes positive (red) or negative (green) correlations. (**C**,**D**) Corresponding co-occurrence (**C**) and correlation (**D**) networks of seed endophytic fungal genera.

Based on relative abundance data at the genus level, bacterial co-occurrence relationships were assessed. The results showed that 32% of the genera were associated with only one region, indicating substantial compositional differences among microbial communities across the four systems. Only 3% of key genera—*Klebsiella*, *Lelliottia*, and *Pseudomonas*—were associated with all four regions. Approximately 10% of the dominant genera were significantly associated with GX, JX, and SD, but not with JS, indicating higher similarity in bacterial composition among the three non-JS regions (Figure 4A).

To identify ecologically important taxa within the *P. massoniana* seed endophytic microbiota, correlation-based network was constructed using the 100 most abundant bacterial genera. Nodes with fewer than one connection were removed, resulting in a network comprising 45 nodes and 59 edges, of which 54 were positive and 4 were negative correlations. Modularity analysis identified 13 distinct modules, indicating a compartmentalized network structure (Figure 4B). Genus-level Zi and Pi values were computed to define the topological roles of each genus. One genus (*SWB02*) exhibited a Zi value greater than 2.5 and was identified as a keystone taxon. In addition, genera with Zi < 2.5 and Pi > 0.62 were classified as connector taxa, including *unclassified_o__Enterobacterales*, *unclassified_k__norank_d__Bacteria*, *Acholeplasma*, *Candidatus_Actinomarina*, *unclassified_f__Flavobacteriaceae*, *Klebsiella*, *unclassified_f__Enterobacteriaceae*, *Alteromonas*, *Marinobacter*, *Prevotella*, *Vibrio*, *Bifidobacterium*, and *Hyphomicrobium*. All remaining genera exhibited low Zi and Pi values and were assigned to peripheral positions within the network.

#### 3.3.2. Fungal Co-Occurrence and Correlation Network

Based on relative abundance data at the genus level, fungal co-occurrence relationships were assessed. The results showed that 23% of the genera were associated with only one region, with no region-specific key genera identified in JX. Only 2% of the dominant genera—*Aspergillus* and *Apiotrichum*—were shared across all four regions. The remaining genera exhibited relatively even distribution patterns across regions (Figure 4C).

To identify ecologically important taxa within the *P. massoniana* seed endophytic microbiota, a correlation-based network was constructed using the 100 most abundant fungal genera. Nodes with fewer than one connection were removed, resulting in a network comprising 32 nodes and 48 edges, including 47 positive and 1 negative correlation. Modularity analysis identified 10 distinct modules, indicating a compartmentalized network structure (Figure 4D). Genus-level Zi and Pi values were computed to define the topological roles of each genus. No genera exhibited Zi values greater than 2.5, indicating the absence of keystone taxa in the fungal network based on Zi–Pi criteria. In contrast, genera with Zi < 2.5 and Pi > 0.62 were classified as connector taxa, including *Phomopsis*, *Mrakia*, *Apiotrichum*, *unclassified_o__Saccharomycetales*, *Penicillium*, *Thelonectria*, *unclassified_f__Ophiostomataceae*, *Cladophialophora*, *Neopestalotiopsis*, *Naganishia*, *unclassified_o__Chaetothyriales*, *Articulospora*, and *unclassified_f__Didymellaceae*. All remaining genera exhibited low Zi and Pi values and were assigned to peripheral positions within the network.

### 3.4. Functional Prediction of Microbial Taxa Shared Between Seeds and Adult P. massoniana

#### 3.4.1. Predicted Functional Profiles of Shared Bacterial Taxa

**Figure 5:**
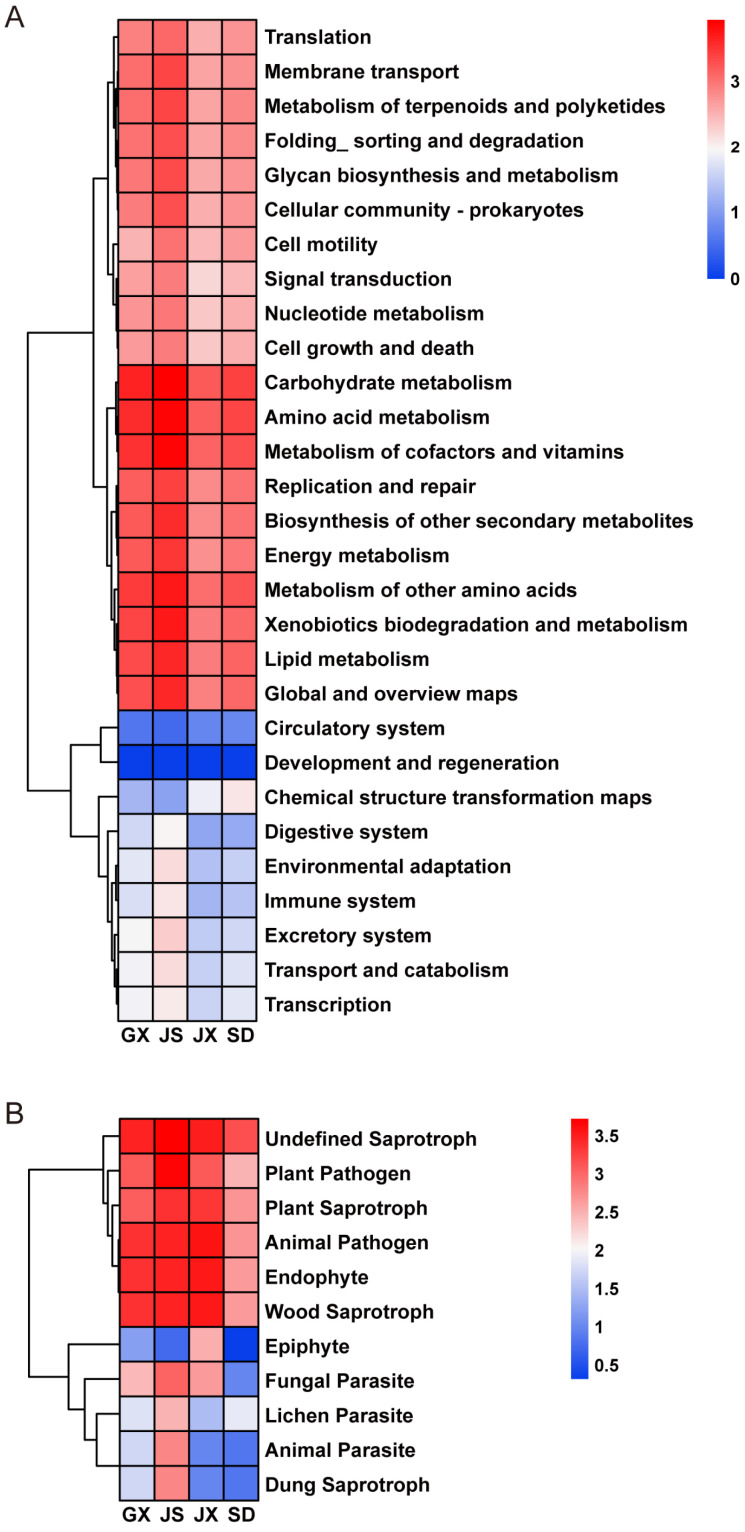
Functional predictions of seed endophytic microbial taxa shared between seeds and adult *P. massoniana*. (**A**) Predicted metabolic functions of shared bacterial taxa based on PICRUSt2, annotated at KEGG pathway level 2. The heatmap shows the relative abundances of major functional categories across samples. (**B**) Proportional distribution of ecological functions of shared fungal taxa predicted by FUNGuild.

Based on the community composition and co-occurrence network analyses, a subset of microbial taxa consistently present in both seeds and adult *P*. *massoniana* trees was selected for further functional prediction. The list of bacterial and fungal taxa shared between seeds and adult trees and used for functional prediction is provided (Table S3). Functional profiling of the shared bacterial communities was performed using PICRUSt2, and the predicted functions were annotated against the Kyoto Encyclopedia of Genes and Genomes (KEGG) database (Figure 5A).

At KEGG level 2, the predicted bacterial functions were mainly associated with metabolism of terpenoids and polyketides, biosynthesis of other secondary metabolites, carbohydrate metabolism, amino acid metabolism, metabolism of cofactors and vitamins, and energy metabolism. Additional functional categories included xenobiotics biodegradation and metabolism, cellular community—prokaryotes, membrane transport, signal transduction, and lipid metabolism. In contrast, pathways related to circulatory system as well as development and regeneration were predicted at relatively low abundances.

#### 3.4.2. Predicted Ecological Functions of Shared Fungal Taxa

Similarly, fungal taxa shared between seeds and adult *P. massoniana* were subjected to functional prediction analysis. FUNGuild-based functional annotation revealed that, across all four regions, the dominant predicted functional groups of the shared fungal communities included undefined saprotroph, plant pathogen, plant saprotroph, animal pathogen, endophyte, and wood saprotroph, with saprotrophy representing the primary nutritional mode (Figure 5B). In contrast, epiphyte, fungal parasite, lichen parasite, animal parasite, and dung saprotroph were predicted at relatively low proportions.

## 4. Discussion

### 4.1. Microbial Composition and Core Symbiotic Taxa of P. massoniana Seeds

In the present study, the dominant bacteria in *P. massoniana* seeds belonged to the phylum Pseudomonadota, particularly the class Gammaproteobacteria, including genera such as *Klebsiella*, *unclassified_o_Enterobacterales*, *Pseudomonas*, *Lelliottia*, *Rahnella1*, and *norank_f_Pectobacteriaceae*. Other major groups included *Lactobacillus* (phylum Bacillota, class Bacilli), and *Microbacterium*, *Gordonia*, and *Branchiibius* (phylum Actinobacteriota, class Actinobacteria). Quantitatively, Pseudomonadota (62.84%) was the most abundant bacterial phylum, followed by Bacillota (13.57%), Bacteroidota (9.04%), and Actinobacteriota (8.53%). This taxonomic profile is broadly consistent with previous seed endophytic microbiota studies. For example, *Microbacterium* was enriched in normal rice seedlings [37]. Similarly, a meta-analysis identified 131 bacterial genera from seeds of 25 common plant species, with the majority belonging to Pseudomonadota, especially Gammaproteobacteria [38], while Actinobacteriota, Bacillota, and Bacteroidota were less prevalent [39]. Similar patterns have been reported in tobacco seeds, where bacteria were dominated by Pseudomonadota and Bacillota [40], and Pseudomonadota, Actinobacteriota, Bacillota, and Bacteroidota were found to be common dominant phyla [41]. The dominant fungal genera in *P. massoniana* seeds were affiliated with the phylum Ascomycota, including *Aspergillus* and *Penicillium* (class Eurotiomycetes), and *Phomopsis*, *unclassified_o_Diaporthales*, *Pestalotiopsis*, and *Diaporthe* (class Sordariomycetes). In the phylum Basidiomycota, dominant genera included *Schizophyllum*, *unclassified_f_Peniophoraceae*, *Sistotrema* (class Agaricomycetes), and *Apiotrichum* (class Tremellomycetes). Quantitatively, Ascomycota was the dominant phylum (66.24%), followed by Basidiomycota (33.35%). The most abundant genera included *Aspergillus* (10.34%), *Trichoderma* (4.75%), and *Penicillium* (2.57%), a pattern also observed in other plant seeds. For instance, the seed endophytic fungal communities of *Gastrodia elata* were dominated by Ascomycota, with *Trichoderma*, *Aspergillus*, and *Penicillium* being prevalent [20]. Taken together, the endophytic microbial communities of *P. massoniana* seeds appears to follow a broadly conserved pattern observed across diverse plant species.

Based on network analysis, a higher proportion of positive edges was observed in the fungal network than in the bacterial network (positive edge ratios: bacteria, 91.53%; fungi, 97.92%), suggesting that a greater proportion of seed endophytic fungi may engage in cooperative or mutually facilitative interactions. In contrast, the bacterial community may harbor relatively more competitive or antagonistic relationships. Additionally, under an equal node threshold, 45 bacterial nodes were associated with other genera, compared with 32 nodes in the fungal network. The bacterial network also exhibited a higher total number of edges, indicating a more complex interaction network among bacterial taxa in *P. massoniana* seeds. In the bacterial network, the presence of a keystone taxon (*SWB02*) together with several connector genera indicates distinct ecological strategies among seed endophytic bacteria. Keystone taxa, characterized by high within-module connectivity, are likely important for maintaining network stability and core functions [42], whereas connector taxa such as *Klebsiella*, *Alteromonas*, and *Prevotella* may promote interactions among different modules [36,43]. Together, these taxa contribute to the structural robustness of the bacterial seed endophytic microbiota. In contrast, no keystone taxa were detected in the fungal network; instead, several fungal genera—including *Phomopsis*, *Penicillium*, *Apiotrichum*, *Thelonectria*, and *Cladophialophora*—were identified as connectors, suggesting that cross-module interactions play a more prominent role in structuring seed endophytic fungal communities. The predominance of connector rather than keystone fungi likely reflects the selective and spatially constrained endophytic seed microhabitat, which favors cooperative, niche-sharing interactions [44]. Collectively, these results characterize the overall structural features of bacterial and fungal networks in *P*. *massoniana* seeds. Given the compositional nature of amplicon sequencing data, correlation-based network inference is inevitably subject to potential closure effects. As a result, the inferred associations should be primarily interpreted as statistical co-occurrence patterns among taxa rather than direct ecological interactions. Nevertheless, in this study, network analysis was mainly employed as an exploratory approach to compare shifts in association patterns across different community contexts and to highlight key taxa that may play important organizational roles within the seed endophytic microbiota.

### 4.2. Putative Vertically Transmitted Microbiota and Their Potential Disease-Resistance Functions

The overlap between microbial taxa in seeds and those found in *P. massoniana* plant tissues supports the notion that microbes are acquired not only via horizontal transmission (e.g., from air, water, or insects) but also through putative vertical transmission from the mother plant. Prior studies on phyllosphere microorganisms have shown that bacterial communities in *P. massoniana* are dominated by Pseudomonadota and Bacillota, with *Lactobacillus* being particularly abundant, while fungal communities are dominated by Ascomycota and Basidiomycota, including genera such as *Cladosporium* and *Pestalotiopsis* [45,46]. In other tissues of *P. massoniana*, commonly detected bacterial genera include *Erwinia*, *Pseudoxanthomonas*, *Brevundimonas*, *Pseudomonas*, *Enterobacter*, and *Klebsiella* which have been isolated from both healthy and diseased tissues [47,48,49]. *Bacillus* has also been detected in *P. massoniana* deadwood [50]. Among these, *Pseudomonas* has repeatedly been shown to play key ecological roles during pine development [51,52]. Fungal taxa such as *Trichoderma*, *Fusarium*, *Alternaria*, *Penicillium*, *Didymella*, *Aspergillus*, *Apiotrichum*, and *Filobasidium* are also frequently reported across pine tissues [47,48,49], with classes such as Agaricomycetes and Sordariomycetes detected in deadwood [14], and belonging to phyla including Ascomycota, Basidiomycota, and Mucoromycota [53]. In our study, many of these taxa were also detected in seed samples, suggesting stable and possibly heritable colonization patterns. Among these, the genera with relatively high abundance included *Klebsiella* (15.55%), *Lactobacillus* (6.42%), *unclassified_o_Enterobacterales* (4.96%), and *Aspergillus* (10.34%), whereas *Pseudomonas* (3.40%) and *Apiotrichum* (4.07%) were present at lower but consistent abundances across all regions. Additional bacterial genera (*Erwinia*, *Brevundimonas*, *Bacillus*) and fungal genera (*Trichoderma*, *Pestalotiopsis*, *Fusarium*, *Alternaria*, *Penicillium*, *Didymella*) were likewise present in seeds. Based on the above findings, we infer that a similar putative vertical transmission mechanism may operate between *P. massoniana* mother trees and their seeds, allowing certain microbial taxa to be inherited across generations.

We further performed functional prediction analyses on selected bacterial genera (*Lactobacillus*, *Erwinia*, *Brevundimonas*, *Pseudomonas*, *Enterobacter*, *Klebsiella*, and *Bacillus*) and fungal genera (*Cladosporium*, *Pestalotiopsis*, *Trichoderma*, *Fusarium*, *Alternaria*, *Penicillium*, *Didymella*, *Aspergillus*, *Apiotrichum*, and *Filobasidium*). The predicted functions of the bacterial taxa were primarily enriched in pathways related to metabolism of terpenoids and polyketides, biosynthesis of other secondary metabolites, carbohydrate metabolism, amino acid metabolism, metabolism of cofactors and vitamins, and energy metabolism. Among these pathways, the enrichment of metabolism of terpenoids and polyketides may indicate potential contributions to resistance against PWD through multiple mechanisms. First, it is functionally connected with phenylpropanoid metabolism, providing precursors for lignin biosynthesis, which reinforces cell walls and strengthens physical barriers against nematode invasion [54]. Second, it is involved in the production of defensive terpenoids such as monoterpenes and diterpenes, which constitute major components of pine resin and exert direct toxicity or inhibitory effects on *B. xylophilus* and its associated fungi [55]. Third, polyketide-derived secondary metabolites can function as antimicrobial compounds or signaling molecules, suppressing the growth of pathogenic fungi and thereby indirectly reducing nematode virulence. FUNGuild-based trophic mode prediction indicated that saprotrophs dominated the fungal communities of *P. massoniana* seeds. Saprotrophic fungi are known for their capacity to degrade lignin and cellulose, thereby contributing to litter decomposition and improving nutrient cycling in forest soils [56,57]. In addition to their role in nutrient turnover, wood decomposition processes may alter the physical and chemical properties of woody tissues, potentially reducing habitat suitability for PWN. Similar ecological dynamics have been reported in insect–fungus systems, where early colonization by *Hylurgops palliatus* and *Monochamus sutor* influenced wood-decaying fungal communities and subsequently affected the establishment of later-arriving species [58]. Notably, a subset of the identified fungal genera was annotated as ectomycorrhizal–wood saprotrophs. These fungi are known to promote host plant growth, enhance soil nutrient availability—particularly phosphorus—and modulate rhizosphere microbial community composition. Such effects may indirectly suppress nematode proliferation. It should be noted that these potential functions are inferred from functional annotation and ecological prediction rather than being experimentally validated, and are further discussed below in the context of previously confirmed studies.

Microbial communities contribute substantially to plant stress tolerance [59,60]. Among the microbial taxa identified in the functional prediction analyses, the bacterial genera *Pseudomonas* and *Bacillus*, as well as the fungal genus *Trichoderma*, have been well documented in previous studies for their roles in PWD control through nematicidal activity, enhancement of host resistance, or promotion of plant growth. For instance, *Pseudomonas abietaniphila* BHJ04 enhances shoot and root growth, restricts PWD progression, and activates defense-related genes in *P. massoniana* [61]. Similarly, *Pseudomonas koreensis* IRP7 reduces disease severity and increases the abundance of beneficial microbial taxa [62], while cell-free culture filtrates of *Pseudomonas aeruginosa* exhibit strong nematicidal activity against the PWN [63]. In addition, *Pseudomonas* species possess multiple nutrient-mobilizing traits, including nitrogen fixation, phosphate solubilization, and siderophore production, which can indirectly strengthen host resistance [64,65,66,67]. *Bacillus* species also exhibit pronounced antagonistic effects against PWN [68,69]. In particular, *Bacillus thuringiensis* produces toxic proteins that damage nematode intestinal tissues, resulting in high mortality rates [70]. *Bacillus amyloliquefaciens* also enhances defense enzyme activities and suppresses nematode infection [71]. Among fungi, *Trichoderma* FXY7, has been shown to significantly inhibit PWN population growth [49]. Other species, including *Trichoderma hamatum* (strain T28), display strong nematicidal effects [72,73], which are largely attributed to the induction of host defense responses and the production of hydrolytic enzymes, antibiotics, and volatile compounds [74,75,76].

Overall, these endophytic microbial taxa from *P. massoniana* seeds—particularly *Pseudomonas*, *Bacillus*, and *Trichoderma*—exhibit functional traits related to nematode suppression, host defense enhancement, and plant growth promotion, and when considered in the context of their potential for putative vertical transmission, provide potential support for the future utilization of endophytic microbiota from *P. massoniana* in PWD research.

### 4.3. Regional Variation in Seed Endophytic Microbial Diversity and Its Implications for PWD

Environmental factors can alter the structure of microbial communities [77]. Our results showed that seed endophytic microbial communities of *P. massoniana* from four geographically distinct regions exhibited a pattern of being “similar but distinct”. At the bacterial level, neither alpha diversity nor richness differed significantly among regions, suggesting relatively stable bacterial diversity across locations. Fungal communities, in contrast, showed higher regional variability. Significant differences in fungal richness were detected between JS and GX as well as SD, indicating that seed endophytic fungi may be more responsive to environmental variation. However, beta diversity differed significantly among regions for both bacterial and fungal communities, with PCoA and permutation-based tests (PERMANOVA and ANOSIM) indicating clear geographic structuring of the seed endophytic microbiota. Indeed, environmental pressures have been shown to shape *P. massoniana* rhizosphere microbial composition [15]. This observation is consistent with previous studies on *P. massoniana* rhizosphere microbiota, where microbial functional traits, such as carbon metabolic activity, were found to vary significantly among provenances and were strongly influenced by soil properties including SOC, TN, and pH, highlighting the role of local environment in shaping microbial traits [78]. This pattern provides a conceptual basis for considering whether modulation of seed endophytic microbial communities could be explored as a possible approach to influence host performance, including disease resistance, in future studies.

Interestingly, the differences in seed endophytic microbial communities of *P. massoniana* across the four regions may be related to the geographical distribution of PWD and variation in host resistance. Previous studies have shown that *P. massoniana* genotypes originating from higher latitudes tend to exhibit lower PWD resistance and growth potential, with both traits increasing from north to south [79]. This pattern aligns with the enrichment of *Lelliottia* in GX samples in our study—a genus known for nitrogen fixation, phosphate solubilization (both organic and inorganic), and IAA production [80]—which may enhance seed germination and early-stage resistance. Furthermore, the widely reported biocontrol fungus *Trichoderma* produces a suite of bioactive compounds, including signaling molecules, antioxidant enzymes, hydrolases, detoxification enzymes, and antibiotics, enabling it to induce systemic resistance and directly suppress PWN. However, our data show that *Trichoderma* was nearly absent in SD samples, potentially reduced contribution to disease suppression in this region. Notably, SD, the region with the lowest average temperature among the four, harbored the highest abundance of *Pseudomonas*, consistent with its reported cold tolerance and ability to support seedling resilience, suggesting that environmental conditions may drive adaptive evolution of *P. massoniana* seeds [81]. Overall, there appears to be a correspondence between the composition and functional attributes of seed endophytic microbial communities and the observed host resistance patterns, possibly reflecting the outcome of long-term coevolutionary adaptation of microbial communities to local pathogen pressure.

## 5. Conclusions

This study revealed the composition and main characteristics of the seed endophytic microbial communities of *P*. *massoniana*. Alpha and beta diversity analysis indicated that seed endophytic microbial communities of *P*. *massoniana* differed among regions, suggesting they may be influenced by environmental factors. Correlation network analysis showed that fungal networks exhibited a higher proportion of positive correlations, whereas bacterial networks were more complex. In addition, we found that taxa such as *Pseudomonas*, *Bacillus*, and *Trichoderma* were present not only in *P. massoniana* seeds but also in mature *P. massoniana* tissues, suggesting a putative vertical transmission. Further functional prediction revealed that these taxa were enriched in pathways related to terpenoid and polyketide metabolism as well as saprotrophic functions. These pathways have been reported to play roles in PWD management. Previous studies have demonstrated the potential of these taxa for the control of PWD. Overall, these findings advance our understanding of seed endophytic microbiota in *P. massoniana* and suggest that targeted manipulation of seed endophytic microbiota may facilitate the development of “immune seedling cultivation” strategies and support further research on PWD.

## Data Availability

The data presented in this study are available in the [Figshare] [10.6084/m9.figshare.29560964].

## Supplementary Materials

The following supporting information can be downloaded at: https://www.mdpi.com/article/10.3390/microorganisms14010199/s1, Figure S1: Representative photographs of *Pinus massoniana* seeds collected from the four sampling regions; Table S1: Detailed list of bacterial genera corresponding to each section of the Venn diagram; Table S2: Detailed list of fungal genera corresponding to each section of the Venn diagram; Table S3: Bacterial and fungal genera used for functional prediction.

## References

[1] Nelson E.B. (2004). Microbial dynamics and interactions in the spermosphere. Annu. Rev. Phytopathol..

[2] Nelson E.B. (2018). The seed microbiome: Origins, interactions, and impacts. Plant Soil.

[3] Shade A., Jacques M.-A., Barret M. (2017). Ecological patterns of seed microbiome diversity, transmission, and assembly. Curr. Opin. Microbiol..

[4] Walitang D.I., Kim C.-G., Jeon S., Kang Y., Sa T. (2019). Conservation and transmission of seed bacterial endophytes across generations following crossbreeding and repeated inbreeding of rice at different geographic locations. MicrobiologyOpen.

[5] Liu L., Liu Y., Qiu F.B., Zhang X.X., Song W. (2008). Advances of studies on micro-ecology in the spermosphere. Microbiology.

[6] Hone H., Mann R., Yang G., Kaur J., Tannenbaum I., Li T., Spangenberg G., Sawbridge T. (2021). Profiling, Isolation and characterisation of beneficial microbes from the seed microbiomes of drought tolerant wheat. Sci. Rep..

[7] Hubbard M., Germida J., Vujanovic V. (2012). Fungal endophytes improve wheat seed germination under heat and drought stress. Botany.

[8] Verma S.K., Kingsley K., Irizarry I., Bergen M., Kharwar R.N., White J.F. (2017). Seed-vectored endophytic bacteria modulate development of rice seedlings. J. Appl. Microbiol..

[9] Wang X., He S.W., Hou J.W., Wei H.L., Zhang X.X. (2023). Advances in seed endophytic bacteriome. Acta Microbiol. Sin..

[10] Ganley R.J., Newcombe G. (2006). Fungal endophytes in seeds and needles of *Pinus monticola*. Mycol. Res..

[11] Cankar K., Kraigher H., Ravnikar M., Rupnik M. (2005). Bacterial endophytes from seeds of Norway spruce (*Picea abies* L. Karst). FEMS Microbiol. Lett..

[12] Doronina N.V., Ivanova E.G., Suzina N.E., Trotsenko Y.A. (2004). Methanotrophs and methylobacteria are found in woody plant tissues within the winter period. Microbiology.

[13] Li Y.X., Zhang X.X. (2018). High risk of invasion and expansion of pine wood nematode in middle temperate zone of China. J. Temperate For. Res..

[14] Chen B., Lu H., Luan F.-G., Zhang Z.-L., Zhang J.-T., Liu X.-P. (2025). Variation in microbiota and chemical components within *Pinus massoniana* during initial wood decay. Microorganisms.

[15] Wu Y., Wang H., Peng L., Zhao H., Zhang Q., Tao Q., Tang X., Huang R., Li B., Wang C. (2024). Root-soil-microbiome interaction in the rhizosphere of Masson pine (*Pinus massoniana*) under different levels of heavy metal pollution. Ecotoxicol. Environ. Saf..

[16] Debray R., Herbert R.A., Jaffe A.L., Crits-Christoph A., Power M.E., Koskella B. (2022). Priority effects in microbiome assembly. Nat. Rev. Microbiol..

[17] Bordenstein S.R., Theis K.R. (2015). Host biology in light of the microbiome: Ten principles of holobionts and hologenomes. PLoS Biol..

[18] Theis K.R., Dheilly N.M., Klassen J.L., Brucker R.M., Baines J.F., Bosch T.C.G., Cryan J.F., Gilbert S.F., Goodnight C.J., Lloyd E.A. (2016). Getting the hologenome concept right: An eco-evolutionary framework for hosts and their microbiomes. Msystems.

[19] Sahu P.K., Tilgam J., Mishra S., Hamid S., Gupta A., K J., Verma S.K., Kharwar R.N. (2021). Surface sterilization for isolation of endophytes: Ensuring what (not) to grow. J. Basic Microbiol..

[20] Xu M.J., Zhou L.X., Wang W.H., He H.Y., Li J.H., Shen M.X., Shen K.Z. (2025). Screening and diversity assessment of endophytic and rhizosphere soil fungi at different seed formation stages of *Gastrodia elata*. Microbiol. China.

[21] Yeates C., Gillings M.R., Davison A.D., Altavilla N., Veal D.A. (1998). Methods for microbial DNA extraction from soil for PCR amplification. Biol. Proced. Online.

[22] Shi S., Kumar S., Young S., Maclean P., Jauregui R. (2023). Evaluation of 16S rRNA gene primer pairs for bacterial community profiling in an across soil and ryegrass plant study. J. Sustain. Agric. Environ..

[23] White T.J., Bruns T.D., Lee S.B., Taylor J.W., Innis M.A., Gelfand D.H., Sninsky J.J., White T.J. (1990). Amplification and direct sequencing of fungal ribosomal RNA genes for phylogenetics. PCR Protocols: A Guide to Methods and Applications.

[24] Caporaso J.G., Lauber C.L., Walters W.A., Berg-Lyons D., Huntley J., Fierer N., Owens S.M., Betley J., Fraser L., Bauer M. (2012). Ultra-high-throughput microbial community analysis on the Illumina HiSeq and MiSeq platforms. ISME J..

[25] Chen S., Zhou Y., Chen Y., Gu J. (2018). Fastp: An ultra-fast all-in-one FASTQ preprocessor. Bioinformatics.

[26] Magoč T., Salzberg S.L. (2011). FLASH: Fast length adjustment of short reads to improve genome assemblies. Bioinformatics.

[27] Wang J., Ma T., Zhao L., Lv J., Li G., Liang F., Liu R. (2008). PCR–DGGE method for analyzing the bacterial community in a high temperature petroleum reservoir. World J. Microbiol. Biotechnol..

[28] Edgar R.C. (2013). UPARSE: Highly accurate OTU sequences from microbial amplicon reads. Nat. Methods.

[29] Wang Q., Garrity G.M., Tiedje J.M., Cole J.R. (2007). Naïve Bayesian classifier for rapid assignment of rRNA sequences into the new bacterial taxonomy. Appl. Environ. Microbiol..

[30] Douglas G.M., Maffei V.J., Zaneveld J.R., Yurgel S.N., Brown J.R., Taylor C.M., Huttenhower C., Langille M.G.I. (2020). PICRUSt2 for prediction of metagenome functions. Nat. Biotechnol..

[31] Nguyen N.H., Song Z., Bates S.T., Branco S., Tedersoo L., Menke J., Schilling J.S., Kennedy P.G. (2016). FUNGuild: An open annotation tool for parsing fungal community datasets by ecological guild. Fungal Ecol..

[32] Han C., Shi C., Liu L., Han J., Yang Q., Wang Y., Li X., Fu W., Gao H., Huang H. (2024). Majorbio Cloud 2024: Update 795 single-cell and multiomics workflows. iMeta.

[33] Schloss P.D., Westcott S.L., Ryabin T., Hall J.R., Hartmann M., Hollister E.B., Lesniewski R.A., Oakley B.B., Parks D.H., Robinson C.J. (2009). Introducing mothur: Open-source, platform-independent, community-supported software for describing and comparing microbial communities. Appl. Environ. Microbiol..

[34] Barberán A., Bates S.T., Casamayor E.O., Fierer N. (2012). Using network analysis to explore co-occurrence patterns in soil microbial communities. ISME J..

[35] Revelle W. (2025). R Package.

[36] Guimerà R., Nunes Amaral L.A. (2005). Functional cartography of complex metabolic networks. Nature.

[37] Nanfack A.D., Nguefack J., Musonerimana S., La China S., Giovanardi D., Stefani E. (2024). Exploiting the microbiome associated with normal and abnormal sprouting rice (*Oryza Sativa* L.) seed phenotypes through a metabarcoding approach. Microbiol. Res..

[38] Truyens S., Weyens N., Cuypers A., Vangronsveld J. (2015). Bacterial seed endophytes: Genera, vertical transmission and interaction with plants. Environ. Microbiol. Rep..

[39] Rosenblueth M., Martínez-Romero E. (2006). Bacterial endophytes and their interactions with hosts. Mol. Plant-Microbe Interact..

[40] Chen X., Krug L., Yang H., Li H., Yang M., Berg G., Cernava T. (2020). *Nicotiana tabacum* seed endophytic communities share a common core structure and genotype-specific signatures in diverging cultivars. Comput. Struct. Biotechnol. J..

[41] Xie H.L., Wang H.C., Cai L.T., Zhou H., Liu C., Lu N., Shi C.H., Wang X.P. (2020). Community structure and diversity of endophytic bacteria of tobacco seeds. Acta Microbiol. Sin..

[42] Banerjee S., Schlaeppi K., van der Heijden M.G.A. (2018). Keystone taxa as drivers of microbiome structure and functioning. Nat. Rev. Microbiol..

[43] de Vries F.T., Griffiths R.I., Bailey M., Craig H., Girlanda M., Gweon H.S., Hallin S., Kaisermann A., Keith A.M., Kretzschmar M. (2018). Soil bacterial networks are less stable under drought than fungal networks. Nat. Commun..

[44] Hodgson S., Cates C., Hodgson J., Morley N.J., Sutton B.C., Gange A.C. (2014). Vertical transmission of fungal endophytes is widespread in forbs. Ecol. Evol..

[45] Yuan G.Y., Sun X.G., Zheng Y., Guo Q.Q., Ding G.J. (2021). Epiphytic microbial diversity of *Pinus massoniana* needles. J. For. Environ..

[46] Deng L., Zheng Y., Sun X.G. (2023). Seasonal characteristics of phyllosphere microbial diversity of *Pinus massoniana*. J. West China For. Sci..

[47] Zhang W., Wang X., Li Y., Liu Z., Li D., Wen X., Feng Y., Zhang X. (2021). Pinewood nematode alters the endophytic and rhizospheric microbial communities of *Pinus massoniana*. Microb. Ecol..

[48] An Y., Li Y., Ma L., Li D., Zhang W., Feng Y., Liu Z., Wang X., Wen X., Zhang X. (2022). The changes of microbial communities and key metabolites after early *Bursaphelenchus xylophilus* invasion of *Pinus massoniana*. Plants.

[49] Wen X.J., Wu J.J., Li Y.X., Wang X., Li Y.X., Zhang X.X. (2022). Microbial diversity analysis of *Pinus massoniana* before and after infected by pine wood nematode. For. Res..

[50] Shi B., Wang X., Yang S., Chen H., Zhao Y., Liu Q., Zou R., Pan Y. (2025). The role of *Pinus massoniana* deadwood in promoting *Plagiomnium acutum* growth: Effects of microbial communities and physicochemical properties. Chem. Biol. Technol. Agric..

[51] Guo Y., Lin Q., Chen L., Carballar-Lejarazú R., Zhang A., Shao E., Liang G., Hu X., Wang R., Xu L. (2020). Characterization of bacterial communities associated with the pinewood nematode insect vector *Monochamus alternatus* hope and the host tree *Pinus massoniana*. BMC Genomics.

[52] Yin S.H., Zhang S.Y., Liu S.S., Wu C.F., Wang J.W., Li Y., Zhang L.Q. (2021). Effect of *Bursaphelenchus xylophilus* infection on the endophytic bacterial community structure in different parts of *Pinus massoniana* seedlings. J. Zhejiang A&F Uni..

[53] Luo X., Yu C. (2021). Diversity of endophytic fungi from *Pinus massoniana* in Guizhou Province, southwestern China. Mycosystema.

[54] Yu O., Jez J.M. (2008). Nature’s assembly line: Biosynthesis of simple phenylpropanoids and polyketides. Plant J..

[55] Liu B., Liu Q., Zhou Z., Yin H., Xie Y., Wei Y. (2020). Two terpene synthases in resistant *Pinus massoniana* contribute to defence against *Bursaphelenchus xylophilus*. Plant Cell Environ..

[56] Ge W., Zhang Z.Y., Dong C.B., Shao Q.Y., Liu Y.X., Han Y.F., Liang Z.Q. (2021). Diversity and functional analysis of the culturable microbes isolated from the fruiting bodies of wild *Cantharellus cibarius*. Mycosystema.

[57] Jia M.H., Wang Z., Liu J.F., Jin M.R., He Z.S., Xing C., Shi Y.W., Shen C.X. (2021). Effects of *Castanopsis kawakamii* forest litter on seed germination of *Pinus massoniana*. For. Res..

[58] Weslien J., Djupström L.B., Schroeder M., Widenfalk O. (2011). Long-term priority effects among insects and fungi colonizing decaying wood. J. Anim. Ecol..

[59] Posada L.F., Arteaga-Figueroa L.A., Adarve-Rengifo I., Cadavid M., Zapata S., Álvarez J.C. (2024). Endophytic microbial diversity associated with commercial cultivar and crop wild relative banana variety could provide clues for microbial community management. Microbiolo. Res..

[60] Wang Z., Yuan J., Wang R., Xu S., Zhou J. (2024). Distinct fungal communities affecting opposite galanthamine accumulation patterns in two *Lycoris* species. Microbiol. Res..

[61] Peng Y., Tang Y., Li D., Ye J. (2024). The growth-promoting and colonization of the pine endophytic *Pseudomonas abietaniphila* for pine wilt disease control. Microorganisms.

[62] Han G., Mannaa M., Kim N., Jeon H.W., Jung H., Lee H.-H., Kim J., Park J., Park A.R., Kim J.-C. (2021). Response of Pine Rhizosphere Microbiota to Foliar Treatment with Resistance-Inducing Bacteria against PineWilt Disease. Microorganisms.

[63] He L.N., Feng Y., Shi H.M., Ye J.R. (2022). Screening and identification of endophytic bacteria with nematicidal activity against *Bursaphelenchus xylophilus* in *Pinus massoniana*. Biotechnol. Bull..

[64] Schenk P.M., Carvalhais L.C., Kazan K. (2012). Unraveling plant–microbe interactions: Can multi-species transcriptomics help?. Trends Biotechnol..

[65] Tahir M., Naeem M.A., Shahid M., Khalid U., Farooq A.B.U., Ahmad N., Ahmad I., Arshad M., Waqar A. (2020). Inoculation of *pqqE* gene inhabiting *Pantoea* and *Pseudomonas* strains improves the growth and grain yield of wheat with a reduced amount of chemical fertilizer. J. Appl. Microbiol..

[66] Ge M.M., Bo Y.L., Liu C., Liu F.C., Xie X.X., Dong Y.L., Ren L.Y. (2023). Screening of soil siderophore-producing bacteria and their activation and utilization of iron oxide. Microbiol. China.

[67] Deepa N., Chauhan S., Singh A. (2024). Unraveling the functional characteristics of endophytic bacterial diversity for plant growth promotion and enhanced secondary metabolite production in *Pelargonium graveolens*. Microbiol. Res..

[68] Ponpandian L.N., Rim S.O., Shanmugam G., Jeon J., Park Y.-H., Lee S.-K., Bae H. (2019). Phylogenetic characterization of bacterial endophytes from four *Pinus* species and their nematicidal activity against the pine wood nematode. Sci. Rep..

[69] Li D., Li Y., Wang X., Zhang W., Wen X., Liu Z., Feng Y., Zhang X. (2023). Engineered pine endophytic *Bacillus toyonensis* with nematocidal and colonization abilities for pine wilt disease control. Front. Microbiol..

[70] Guo Y., Weng M., Sun Y., Carballar-Lejarazú R., Wu S., Lian C. (2022). *Bacillus thuringiensis* toxins with nematocidal activity against the pinewood nematode *Bursaphelenchus xylophilus*. J. Invertebr. Pathol..

[71] Zhu J., Deng C., Zhang Y., Liu M., Zhou G., Liu J. (2025). Pine rhizosphere soil microorganisms enhance the growth and resistance of *Pinus massoniana* against nematode infection. Microorganisms.

[72] Wei B.H., Zhang K., Shen J.X., Tang H., Song R.Q. (2017). The insecticidal activities of ethyl acetate efficiently extracts from fifteen bio-control strains to pine wood nematode. For. Sci. Technol..

[73] Tian Z.L. (2016). Isolation, Identification and Application of Fungal Biocontrol Agents to *Heterodera glycines*. Master’s Thesis.

[74] Yuan W.T. (2011). The Preliminary Research on the Flora of Endophytic Bacteria of Pine and Indoor Screening of Biocontrol Bacteria. Master’s Thesis.

[75] Yang Z., Yu Z., Lei L., Xia Z., Shao L., Zhang K., Li G. (2012). Nematicidal effect of volatiles produced by *Trichoderma* sp. J. Asia-Pac. Entomol..

[76] Jiao N., Yin D.C., Song R.Q. (2021). Transcriptome study on the disease resistance related genes induced by *Bursaphelenchus xylophilus* in *Trichoderma asperellum* T203. J. Shenyang Agric. Univ..

[77] Ma C., Du P., Cao Y., Liu H., Ma L., Liang B. (2024). Melatonin alleviates apple replant disease by regulating the endophytic microbiome of roots and phloridzin accumulation. Microbiol. Res..

[78] Huang Z., Qin Y., He X., Zhang M., Ren X., Yu W., Ji K. (2024). Analysis on metabolic functions of rhizosphere microbial communities of *Pinus massoniana* provenances with different carbon storage by Biolog eco microplates. Front. Microbiol..

[79] Xu F.Y., Ge M.H., Wang Q.M., Zhang P., Zhu K.G., Zhao J.L., Xu D., Wang M.M. (1998). Studies on the *Masson* pine provenances resistance to pine wood nematode (PWN) disease in China. J. Nanjing For. Univ..

[80] Yang M., Gao T., Li Y.J., Wei C.Y., Gao M., Ma L.J. (2020). Isolation and screening of plant growth-promoting rhizobacteria in pepper and their disease-resistant growth-promoting characteristics. Biotechnol. Bull..

[81] Marian M., Antonielli L., Pertot I., Perazzolli M. (2025). Amplicon sequencing and culture-dependent approaches reveal core bacterial endophytes aiding freezing stress tolerance in alpine Rosaceae plants. mBio.

