# Supplementary material for: Characterization of Seed Endophytic Microbiota in Pinus massoniana
Source: Microorganisms. 2026 Jan 15;14(1):199. doi: 10.3390/microorganisms14010199 (PMC12843720; doi:10.3390/microorganisms14010199)
Supplement: Supplementary file 1 [file microorganisms-14-00199-s001.zip › microorganisms-4080871-supplementary.pdf]

Figure S1. Representative photographs of *Pinus massoniana* seeds collected from the four sampling regions.

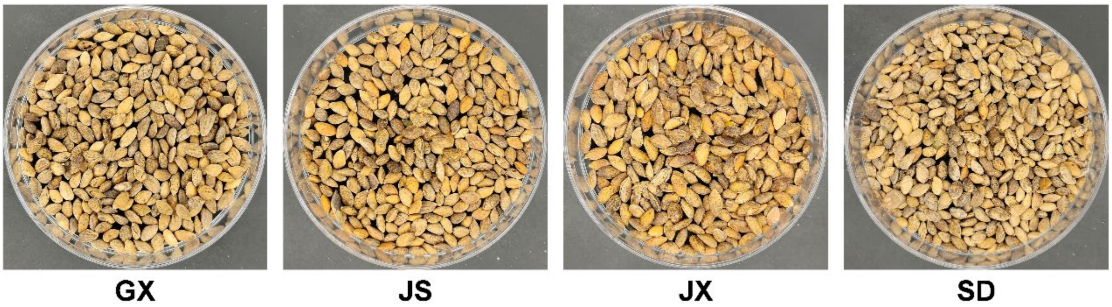

Table S1. Detailed list of bacterial genera corresponding to each section of the Venn diagram.

Venn Section	Genera
GX only (27)	*Georgenia*<br>*Paenarthrobacter*<br>*Ruminofilibacter*<br>*Filimonas*<br>*Ignavibacterium*<br>*BBMC-4*<br>*Oxobacter*<br>*28-4*<br>*Dorea*<br>*Fusicatenibacter*<br>*Tyzzerella*<br>*Hungateiclostridium*<br>*norank_f__norank_o__Saccharimonadales*<br>*norank_f__WD2101_soil_group*<br>*Asticcacaulis*<br>*Neo-b11*<br>*Pleomorphomonas*<br>*Pseudorhodoplanes*<br>*Amantichitinum*<br>*Aquitalea*<br>*Alicycliphilus*<br>*Duganella*<br>*Dechloromonas*<br>*unclassified_f__Rhodocyclaceae*<br>*norank_f__norank_o__CCD24*<br>*Candidatus_Metachlamydia*<br>*norank_f__norank_o__WCHB1-41*
JS only (165)	*Acanthopleuribacter*<br>*norank_f__norank_o__norank_c__Subgroup_22*<br>*norank_f__norank_o__Actinomarinales*<br>*norank_f__norank_o__norank_c__Acidimicrobiia*<br>*Mobiluncus*<br>*Varibaculum*<br>*unclassified_f__Frankiaceae*<br>*norank_f__norank_o__Frankiales*<br>*Oryzihumus*<br>*unclassified_f__Micromonosporaceae*<br>*Aestuariimicrobium*<br>*Actinomycetospora*<br>*Thermotunica*<br>*Actinomadura*<br>*Coriobacteriaceae_UCG-002*

	norank_f__Bacteroidales_BS11_gut_group
	norank_f__Bacteroidales_UCG-001
	norank_f__Bacteroidetes_BD2-2
	Petrimonas
	norank_f__F082
	Alloprevotella
	Roseimarinus
	Lewinella
	unclassified_f__Saprospiraceae
	norank_f__Cyclobacteriaceae
	unclassified_f__Crocinitomicaceae
	Owenweeksia
	unclassified_f__Cryomorphaceae
	Aquimarina
	Mesoflavibacter
	Tenacibaculum
	Zunongwangia
	norank_f__Flavobacteriaceae
	Pedobacter
	unclassified_c__Bacteroidia
	Chlorobium
	norank_f__Balneolaceae
	norank_f__Rhodothermaceae
	norank_f__Bacteriovoracaceae
	unclassified_f__Bacteriovoracaceae
	Pseudobacteriovorax
	unclassified_f__norank_o__Oligoflexales
	norank_f__Silvanigrellaceae
	norank_f__AKIW781
	norank_f__norank_o__Dehalococcoidales
	norank_f__norank_o__S085
	norank_f__norank_o__SAR202_clade
	norank_f__Ktedonobacteraceae
	norank_f__Vermiphilaceae
	unclassified_o__Babeliales
	norank_f__Desulfobulbaceae
	Desulforhopalus
	norank_f__Desulfocapsaceae
	Bilophila
	norank_f__norank_o__Bradymonadales
	unclassified_p__Desulfobacterota
	norank_f__Fibrobacteraceae
	Acholeplasma
	Ureibacillus

	Rummeliibacillus<br>*Erysipelothrix*<br>*Leuconostoc*<br>*unclassified_o__Lactobacillales*<br>*Thermoflavimicrobium*<br>*Pseudoramibacter*<br>*Garciella*<br>*Acetitomaculum*<br>*Eubacterium_ruminantium_group*<br>*Lachnospiraceae_NK4A136_group*<br>*UCG-009*<br>*Acetanaerobacterium*<br>*norank_f__Eubacterium_coprostanoligenes_group*<br>*Colidextribacter*<br>*unclassified_f__Oscillospiraceae*<br>*Caproiciproducens*<br>*norank_f__Ruminococcaceae*<br>*unclassified_f__Ruminococcaceae*<br>*Peptococcus*<br>*unclassified_f__Anaerovoracaceae*<br>*Terrisporobacter*<br>*Anaerococcus*<br>*Ezakiella*<br>*Finegoldia*<br>*norank_f__Hungateiclostridiaceae*<br>*Desulfosporosinus*<br>*Pelosinus*<br>*Dialister*<br>*norank_f__norank_o__norank_c__BD2-11_terrestrial_group*<br>*Myxococcus*<br>*norank_f__norank_o__Blfdi19*<br>*norank_f__Sandaracinaceae*<br>*norank_f__norank_o__norank_c__bacteriap25*<br>*Thermodesulfovibrio*<br>*norank_f__norank_o__Candidatus_Brennerbacteria*<br>*norank_f__norank_o__norank_c__OM190*<br>*Belnapia*<br>*Endobacter*<br>*Roseococcus*<br>*norank_f__Acetobacteraceae*<br>*norank_f__norank_o__Azospirillales*<br>*Hyphomonas*<br>*Maricaulis*<br>*Oceanicaulis*

	Amphiplicatus<br>*norank_f__norank_o__Elsterales*<br>*Tagaea*<br>*Kordiimonas*<br>*norank_f__Micavibrionaceae*<br>*norank_f__norank_o__Micavibrionales*<br>*unclassified_o__Micavibrionales*<br>*norank_f__Paracaedibacteraceae*<br>*Candidatus_Phaeomarinobacter*<br>*Pyruvatibacter*<br>*Chelatococcus*<br>*unclassified_f__Hyphomicrobiaceae*<br>*Labrenzia*<br>*Stappia*<br>*Limibaculum*<br>*Lutimaribacter*<br>*Marivita*<br>*Rhodobaculum*<br>*Roseibacterium*<br>*Ruegeria*<br>*Shimia*<br>*Tropicimonas*<br>*unclassified_f__Magnetospirillaceae*<br>*norank_f__Sphingomonadaceae*<br>*OM75_clade*<br>*Sulfurifustis*<br>*Aestuariibacter*<br>*unclassified_f__Alteromonadaceae*<br>*Thalassotalea*<br>*norank_f__Beggiatoaceae*<br>*norank_f__A21b*<br>*Rhizobacter*<br>*Neisseria*<br>*unclassified_f__Cellvibrionaceae*<br>*C1-B045*<br>*Candidatus_Competibacter*<br>*norank_f__Erwiniaceae*<br>*Caedibacter_taeniospiralis_group*<br>*norank_f__norank_o__Ga0077536*<br>*norank_f__unclassified*<br>*norank_f__norank_o__HOC36*<br>*Methylobacter*<br>*AqS1*<br>*Cm1-21*

	norank_f__norank_o__OM182_clade<br>*Ketobacter*<br>*Alcanivorax*<br>*Kangiella*<br>*Marinomonas*<br>*Neptunomonas*<br>*Oleiphilus*<br>*Pseudohongiella*<br>*Haemophilus*<br>*Rodentibacter*<br>*Salinisphaera*<br>*Thaumasiovibrio*<br>*Chujaibacter*<br>*Lysobacter*<br>*norank_f__norank_o__norank_c__Gammaproteobacteria*<br>*Turneriella*<br>*Treponema*<br>*norank_f__Spirochaetaceae*
JX only (99)	*norank_f__norank_o__Acidobacteriales*<br>*norank_f__norank_o__Microtrichales*<br>*Nocardia*<br>*hgcI_clade*<br>*Quadrisphaera*<br>*Janibacter*<br>*Knoellia*<br>*Agromyces*<br>*Amnibacterium*<br>*Mycetocola*<br>*Glutamicibacter*<br>*Kocuria*<br>*norank_f__Nitriliruptoraceae*<br>*Amycolatopsis*<br>*Pseudonocardia*<br>*Streptomonospora*<br>*unclassified_c__Actinobacteria*<br>*norank_f__norank_o__OPB41*<br>*Rubrobacter*<br>*norank_f__67-14*<br>*Patulibacter*<br>*norank_f__Fimbriimonadaceae*<br>*Butyricimonas*<br>*Prevotellaceae_UCG-001*<br>*Rikenella*<br>*unclassified_o__Bacteroidales*

	Pontibacter<br>*Aurantivirga*<br>*Lutibacter*<br>*Planktosalinus*<br>*Bergeyella*<br>*Cloacibacterium*<br>*norank_f__Weeksellaceae*<br>*norank_f__Lentimicrobiaceae*<br>*norank_f__norank_o__0319-6G20*<br>*Kouleothrix*<br>*norank_f__JG30-KF-CM45*<br>*norank_f__JG30-KF-AS9*<br>*norank_f__norank_o__norank_c__TK10*<br>*unclassified_p__Chloroflexi*<br>*unclassified_f__Geobacteraceae*<br>*Tumebacillus*<br>*unclassified_f__Bacillaceae*<br>*Kurthia*<br>*Lysinibacillus*<br>*norank_f__Aerococcaceae*<br>*norank_f__Carnobacteriaceae*<br>*Gemella*<br>*norank_f__norank_o__Clostridia_UCG-014*<br>*norank_f__norank_o__Clostridia_vadinBB60_group*<br>*Fonticella*<br>*Clostridium_sensu_stricto_7*<br>*Defluviitalea*<br>*Eubacterium_eligens_group*<br>*Eubacterium_oxidoreducens_group*<br>*Herbinix*<br>*Lachnoanaerobaculum*<br>*Lachnospiraceae_NC2004_group*<br>*Lachnospiraceae_NK3A20_group*<br>*Marvinbryantia*<br>*Ruminococcus_torques_group*<br>*norank_f__Lachnospiraceae*<br>*Faecalibacterium*<br>*Subdoligranulum*<br>*Sporacetigenium*<br>*W5053*<br>*Lutispora*<br>*HN-HF0106*<br>*Thermoclostridium*<br>*norank_f__Ammonificaceae*

	Dethiobacter<br>*norank_f__Sporomusaceae*<br>*Syntrophomonas*<br>*norank_f__norank_o__norank_c__norank_p__Firmicutes*<br>*Pajaroellobacter*<br>*norank_f__norank_o__norank_c__norank_p__NB1-j*<br>*Caulobacter*<br>*norank_f__Caulobacteraceae*<br>*Bosea*<br>*Hyphomicrobium*<br>*Mesorhizobium*<br>*Bauldia*<br>*Afipia*<br>*Rubellimicrobium*<br>*norank_f__Magnetospirillaceae*<br>*Sandaracinobacter*<br>*Aeromonas*<br>*norank_f__Hydrogenophilaceae*<br>*unclassified_f__Neisseriaceae*<br>*Azoarcus*<br>*Thauera*<br>*norank_f__SC-I-84*<br>*norank_f__Sutterellaceae*<br>*norank_f__TRA3-20*<br>*Coxiella*<br>*Moraxella*<br>*norank_f__Solimonadaceae*<br>*Thiothrix*<br>*Leptonema*
SD only (35)	*Paludibaculum*<br>*CL500-29_marine_group*<br>*Kineosporia*<br>*Cellulomonas*<br>*Leucobacter*<br>*Prevotellaceae_UCG-003*<br>*Haliscomenobacter*<br>*Fluviicola*<br>*Elizabethkingia*<br>*norank_f__KD3-93*<br>*Lentimicrobium*<br>*norank_f__env.OPS_17*<br>*norank_f__norank_o__Kapabacteriales*<br>*unclassified_f__Roseiflexaceae*<br>*norank_f__norank_o__Babeliales*

	norank_f__Syntrophobacteraceae<br>*unclassified_o__Bacillales*<br>*Weissella*<br>*Christensenellaceae_R-7_group*<br>*norank_f__norank_o__norank_c__S0134_terrestrial_group*<br>*Haliangium*<br>*Hirschia*<br>*Pseudochrobactrum*<br>*norank_f__Rhizobiaceae*<br>*unclassified_o__Rhizobiales*<br>*Seohaeicola*<br>*Sphingoaurantiacus*<br>*norank_f__norank_o__norank_c__Alphaproteobacteria*<br>*Pelistega*<br>*unclassified_f__Methylophilaceae*<br>*Ellin6067*<br>*Georgfuchsia*<br>*norank_f__Ectothiorhodospiraceae*<br>*norank_f__norank_o__norank_c__norank_p__RCP2-54*<br>*Thermovirga*
GX & JS only (17)	*unclassified_f__Acidobacteriaceae_Subgroup_1*<br>*IMCC26207*<br>*Paludibacter*<br>*norank_f__norank_o__Chitinophagales*<br>*Aquibacter*<br>*Paenibacillus*<br>*Anaerocolumna*<br>*Propionispira*<br>*Parvularcula*<br>*Micropepsis*<br>*norank_f__Rhizobiales_Incertae_Sedis*<br>*norank_f__AEGEAN-169_marine_group*<br>*Telmatospirillum*<br>*Microvirgula*<br>*Paludibacterium*<br>*Citrobacter*<br>*norank_f__norank_o__norank_c__norank_p__SAR324_cladeMarine_group_B*
GX & JX only (10)	*Kineococcus*<br>*unclassified_f__Dermacoccaceae*<br>*Conexibacter*<br>*Flavobacterium*<br>*Deinococcus*<br>*Haloplasma*<br>*norank_f__UCG-010*

	g__Fastidiosipila<br>*g__Rhizorhapis*<br>*g__Proteus*
GX & SD only (9)	*norank_f__norank_o__IMCC26256*<br>*Micrococcus*<br>*norank_f__norank_o__SJA-15*<br>*norank_f__norank_o__norank_c__norank_p__Desulfobacterota*<br>*TM7a*<br>*Phreatobacter*<br>*unclassified_o__Burkholderiales*<br>*Pasteurella*<br>*unclassified_o__Xanthomonadales*
JS & JX only (48)	*Abditibacterium*<br>*Candidatus_Solibacter*<br>*Sva0996_marine_group*<br>*Modestobacter*<br>*Nakamurella*<br>*Candidatus_Aquiluna*<br>*Curtobacterium*<br>*Arthrobacter*<br>*norank_f__norank_o__PeM15*<br>*Thermobifida*<br>*Solirubrobacter*<br>*Prevotellaceae_NK3B31_group*<br>*Alistipes*<br>*Rikenellaceae_RC9_gut_group*<br>*Peredibacter*<br>*norank_f__norank_o__norank_c__AD3*<br>*Desulfovibrio*<br>*Planococcus*<br>*Sporolactobacillus*<br>*Pediococcus*<br>*Thermobacillus*<br>*Hydrogenibacillus*<br>*Caldicoprobacter*<br>*unclassified_f__Caloramatoraceae*<br>*Clostridium_sensu_stricto_3*<br>*Roseburia*<br>*unclassified_f__Lachnospiraceae*<br>*norank_f__norank_o__Proteinivoracales*<br>*unclassified_f__Hungateiclostridiaceae*<br>*unclassified_c__Clostridia*<br>*norank_f__norank_o__norank_c__D8A-2*<br>*norank_f__norank_o__Limnochordales*

	norank_f__norank_o__M55-D21<br>*P3OB-42*<br>*Devosia*<br>*Rhodoplanes*<br>*Ascidiaceihabitans*<br>*Thalassospira*<br>*Erythrobacter*<br>*MN_122.2a*<br>*norank_f__Alteromonadaceae*<br>*Idiomarina*<br>*Lautropia*<br>*Limnobacter*<br>*Acidovorax*<br>*Luminiphilus*<br>*unclassified_f__Vibrionaceae*<br>*Defluviitoga*
JS & SD only (24)	*Blastococcus*<br>*Marinoscillum*<br>*NS2b_marine_group*<br>*unclassified_f__Flavobacteriaceae*<br>*norank_f__NS7_marine_group*<br>*norank_f__NS9_marine_group*<br>*norank_f__norank_o__PB19*<br>*Sporobacter*<br>*Fusobacterium*<br>*norank_f__norank_o__norank_c__norank_p__Marinimicrobia_SAR406_clade*<br>*norank_f__Hyphomonadaceae*<br>*norank_f__norank_o__Defluviicoccales*<br>*Reyranella*<br>*norank_f__Xanthobacteraceae*<br>*Cribrihabitans*<br>*Litorimicrobium*<br>*norank_f__Rhodospirillaceae*<br>*unclassified_f__Comamonadaceae*<br>*norank_f__MWH-UniP1_aquatic_group*<br>*mle1-7*<br>*Marinobacterium*<br>*Woeseia*<br>*norank_f__norank_o__UBA10353_marine_group*<br>*SCGC_AAA164-E04*
JX & SD only (13)	*norank_f__Roseiflexaceae*<br>*Blautia*<br>*Leptotrichia*<br>*norank_f__Polyangiaceae*

	Phenylobacterium<br>*unclassified_f__Xanthobacteraceae*<br>*Sphingopyxis*<br>*Ideonella*<br>*Nitrosomonas*<br>*Methyloversatilis*<br>*Pantoea*<br>*Acidibacter*<br>*Steroidobacter*
GX & JS & JX only (30)	*unclassified_f__Ilumatobacteraceae*<br>*Gardnerella*<br>*Tsukamurella*<br>*Nocardioides*<br>*Bacteroides*<br>*norank_f__Muribaculaceae*<br>*Porphyromonas*<br>*Prevotella*<br>*Sphingobacterium*<br>*Candidatus_Caldatribacterium*<br>*Staphylococcus*<br>*Clostridium_sensu_stricto_1*<br>*Clostridium_sensu_stricto_12*<br>*Cellulosilyticum*<br>*Lachnoclostridium*<br>*Paeniclostridium*<br>*Tepidimicrobium*<br>*norank_f__Limnochordaceae*<br>*norank_f__norank_o__MBA03*<br>*norank_f__BIrii41*<br>*Aureimonas*<br>*Ensifer*<br>*Bradyrhizobium*<br>*Marinobacter*<br>*unclassified_f__Chromobacteriaceae*<br>*Ottowia*<br>*OM60NOR5_clade*<br>*Escherichia-Shigella*<br>*Yersinia*<br>*Halomonas*
GX & JS & SD only (5)	*Streptomyces*<br>*Balneola*<br>*norank_f__Gemmatimonadaceae*<br>*norank_f__PS1_clade*<br>*Salmonella*

GX & JX & SD only (19)	*Bifidobacterium*<br>*Corynebacterium*<br>*norank_f__Bacteroidetes_vadinHA17*<br>*OLB12*<br>*norank_f__Microscillaceae*<br>*Chloronema*<br>*unclassified_f__Chloroflexaceae*<br>*Phaselicystis*<br>*Nitrospira*<br>*SWB02*<br>*Dongia*<br>*unclassified_f__Beijerinckiaceae*<br>*Pedomicrobium*<br>*Clade_Ia*<br>*SD04E11*<br>*Hydrogenophaga*<br>*MND1*<br>*Denitratisoma*<br>*norank_f__norank_o__JG36-GS-52*
JS & JX & SD only (29)	*Actinomyces*<br>*Acidothermus*<br>*Neomicrococcus*<br>*Rothia*<br>*norank_f__norank_o__norank_c__MB-A2-108*<br>*Gaiella*<br>*norank_f__norank_o__Gaiellales*<br>*norank_f__Cryomorphaceae*<br>*Flavicella*<br>*Chlorobaculum*<br>*OM27_clade*<br>*Exiguobacterium*<br>*Enterococcus*<br>*Ruminococcus*<br>*Peptoniphilus*<br>*Ruminiclostridium*<br>*norank_f__SAR116_clade*<br>*unclassified_f__Rhizobiaceae*<br>*norank_f__S25-593*<br>*Alteromonas*<br>*unclassified_f__Alcaligenaceae*<br>*Leptothrix*<br>*Variovorax*<br>*Thiobacillus*<br>*Kosakonia*

	Litoricola<br>*norank_f__Nitrincolaceae*<br>*norank_f__norank_o__SAR86_clade*<br>*Dyella*
GX & JS & JX & SD only (67)	*Bryobacter*<br>*Candidatus_Actinomarina*<br>*Mycobacterium*<br>*Gordonia*<br>*Rhodococcus*<br>*Branchiibius*<br>*Microbacterium*<br>*Cutibacterium*<br>*norank_f__Saprospiraceae*<br>*Formosa*<br>*NS4_marine_group*<br>*NS5_marine_group*<br>*Chryseobacterium*<br>*norank_f__norank_o__norank_c__SJA-28*<br>*Bdellovibrio*<br>*Bacillus*<br>*Turicibacter*<br>*Granulicatella*<br>*Lactobacillus*<br>*Streptococcus*<br>*Romboutsia*<br>*unclassified_f__Peptostreptococcaceae*<br>*norank_f__Dethiobacteraceae*<br>*norank_f__norank_o__norank_c__Limnochordia*<br>*SM1A02*<br>*Roseomonas*<br>*unclassified_f__Acetobacteraceae*<br>*Brevundimonas*<br>*Methylobacterium-Methylorubrum*<br>*Allorhizobium-Neorhizobium-Pararhizobium-Rhizobium*<br>*norank_f__Stappiaceae*<br>*HIMB11*<br>*Haematobacter*<br>*Paracoccus*<br>*unclassified_f__Rhodobacteraceae*<br>*Novosphingobium*<br>*Sphingobium*<br>*Sphingomonas*<br>*unclassified_c__Alphaproteobacteria*<br>*Rheinheimera*

	Pseudoalteromonas<br>*Burkholderia-Caballeronia-Paraburkholderia*<br>*Pandoraea*<br>*Ralstonia*<br>*Aquabacterium*<br>*Comamonas*<br>*Delftia*<br>*Pelomonas*<br>*Herbaspirillum*<br>*Massilia*<br>*SAR92_clade*<br>*Klebsiella*<br>*Lelliottia*<br>*unclassified_f__Enterobacteriaceae*<br>*unclassified_f__Erwiniaceae*<br>*norank_f__Pectobacteriaceae*<br>*Rahnella1*<br>*unclassified_o__Enterobacterales*<br>*Acinetobacter*<br>*Pseudomonas*<br>*Vibrio*<br>*Luteimonas*<br>*Stenotrophomonas*<br>*unclassified_f__Xanthomonadaceae*<br>*unclassified_c__Gammaproteobacteria*<br>*unclassified_p__Proteobacteria*<br>*unclassified_k__norank_d__Bacteria*

Table S2. Detailed list of fungal genera corresponding to each section of the Venn diagram.

Venn Section	Genera
GX only (40)	*Lasiodiplodia*
	Ramichloridium
	Dothiora
	Cenococcum
	Corynespora
	unclassified_f__Montagnulaceae
	Torula
	Titaea
	unclassified_c__Eurotiomycetes
	Cistella
	Glutinomyces
	unclassified_f__Hyaloscyphaceae
	Hanseniaspora
	Coniochaeta
	Cytospora
	unclassified_f__Amplistromataceae
	Lecanicillium
	Sarocladium
	Dactylonectria
	Volutella
	Myxospora
	Wardomyces
	Phialemonium
	unclassified_f__Chaetomiaceae
	unclassified_f__Lasiosphaeriaceae
	unclassified_o__Sordariales
	Apiospora
	Monosporascus
	Microdochium
	Anthostomella
	Echinoderma
	Simocybe
	Psathyrella
	unclassified_o__Agaricales
	Rhizoctonia
	Colacogloea
	Itersonilia
	Krasilnikovozyma
	Piskurozyma
	Kockovaella
JS only (51)	*Arthrocatena*
	Rachicladosporium

	Toxicocladosporium<br>*Septoria*<br>*unclassified_f__Dothioraceae*<br>*Angustimassarina*<br>*Alloleptosphaeria*<br>*Vaginatispora*<br>*Periconia*<br>*Chaetosphaeronema*<br>*Phaeosphaeria*<br>*Wojnowiciella*<br>*Exserohilum*<br>*Stemphylium*<br>*Capronia*<br>*Sarea*<br>*Cadophora*<br>*Chalara*<br>*Polydesmia*<br>*Hyphozyma*<br>*unclassified_c__Leotiomycetes*<br>*Scheffersomyces*<br>*Yamadazyma*<br>*Nakazawaea*<br>*Ogataea*<br>*Kluyveromyces*<br>*Kuraishia*<br>*Plectosphaerella*<br>*unclassified_f__Cordycipitaceae*<br>*Leptographium*<br>*Phomatospora*<br>*Diatrypella*<br>*Agaricus*<br>*Gymnopus*<br>*Psilocybe*<br>*Heterochaete*<br>*Gloeoporus*<br>*Phanerochaete*<br>*unclassified_f__Serendipitaceae*<br>*unclassified_o__Tremellodendropsidales*<br>*Chionosphaera*<br>*Kurtzmanomyces*<br>*Occultifur*<br>*Buckleyzyma*<br>*Erythrobasidium*<br>*Acaromyces*

	Trigonosporomyces<br>*Sporobolomyces*<br>*Cystofilobasidium*<br>*unclassified_c__GS13*<br>*Powellomyces*
JX only (36)	*unclassified_f__Teratosphaeriaceae*<br>*Dothidea*<br>*Hortaea*<br>*unclassified_o__Dothideales*<br>*Coniothyrium*<br>*Neocucurbitaria*<br>*Paraphaeosphaeria*<br>*Spegazzinia*<br>*Setophaeosphaeria*<br>*Setophoma*<br>*Shiraia*<br>*unclassified_f__Tubeufiaceae*<br>*Hyalocladosporiella*<br>*Strelitziana*<br>*Coniosporium*<br>*Xeromyces*<br>*unclassified_f__Aspergillaceae*<br>*unclassified_c__Lecanoromycetes*<br>*Scytalidium*<br>*Phacidium*<br>*Pseudogymnoascus*<br>*Orbilia*<br>*Wilcoxina*<br>*unclassified_f__Dipodascaceae*<br>*Jattaea*<br>*Engyodontium*<br>*Cortinarius*<br>*Clitopilus*<br>*Coprinellus*<br>*unclassified_f__Stephanosporaceae*<br>*Exidiopsis*<br>*unclassified_o__Auriculariales*<br>*unclassified_o__Phallales*<br>*Serendipita*<br>*Sarcodon*<br>*Trechispora*
SD only (28)	*Botryosphaeria*<br>*Pseudocercospora*<br>*Neodevriesia*

	Paraconiothyrium<br>*Microsphaeropsis*<br>*Roussoella*<br>*Coccodinium*<br>*Cladophialophora*<br>*Alatosessilispora*<br>*Thermoascus*<br>*Pezicula*<br>*Oidiodendron*<br>*Leuconeurospora*<br>*unclassified_f__Metschnikowiaceae*<br>*Saccharomycopsis*<br>*Colletotrichum*<br>*Thelonectria*<br>*Pithoascus*<br>*unclassified_f__Microdochiaceae*<br>*Neopestalotiopsis*<br>*Whalleya*<br>*Xylaria*<br>*Oxyporus*<br>*Antrodia*<br>*unclassified_c__Agaricomycetes*<br>*Slooffia*<br>*unclassified_o__GS24*<br>*Ramicandelaber*
GX & JS only (12)	*Cercospora*<br>*Devriesia*<br>*Rhizosphaera*<br>*Sydowia*<br>*Neocosmospora*<br>*unclassified_f__Nectriaceae*<br>*Hansfordia*<br>*Hygrophorus*<br>*Uncobasidium*<br>*Curvibasidium*<br>*Papiliotrema*<br>*unclassified_f__Neocallimastigaceae*
GX & JX only (10)	*Pseudopithomyces*<br>*Thermomyces*<br>*Debaryomyces*<br>*Saccharomyces*<br>*Chaetomium*<br>*Schizothecium*<br>*Ramophialophora*

	Sterigmatomyces<br>*Vishniacozyma*<br>*unclassified_o__Tremellales*
GX & SD only (3)	*Pseudeurotium*<br>*Zygosporium*<br>*Coprinus*
JS & JX only (19)	*Phaeococcomyces*<br>*unclassified_f__Cucurbitariaceae*<br>*Leptosphaeria*<br>*unclassified_f__Lophiostomataceae*<br>*Neosetophoma*<br>*Setomelanomma*<br>*unclassified_f__Phaeosphaeriaceae*<br>*Sporormiella*<br>*Knufia*<br>*unclassified_f__Trichomeriaceae*<br>*Articulospora*<br>*Thelebolus*<br>*Lectera*<br>*Zalerion*<br>*Piloderma*<br>*Kondoa*<br>*unclassified_c__Cystobasidiomycetes*<br>*Cryptococcus_f__Tremellaceae*<br>*Rhizophlyctis*
JS & SD only (3)	*Cyphellophora*<br>*unclassified_f__Cyphellophoraceae*<br>*unclassified_f__Ophiostomataceae*
JX & SD only (5)	*Archaeorhizomyces*<br>*Sclerostagonospora*<br>*Curvularia*<br>*Acremonium*<br>*Sporidiobolus*
GX & JS & JX only (16)	*Zasmidium*<br>*unclassified_f__Mycosphaerellaceae*<br>*unclassified_f__Didymosphaeriaceae*<br>*Lophiostoma*<br>*unclassified_c__Dothideomycetes*<br>*unclassified_o__Chaetothyriales*<br>*Ascobolus*<br>*Gibberella*<br>*unclassified_f__Ceratobasidiaceae*<br>*Irpex*<br>*Cystobasidium*

	Tausonia<br>*Naganishia*<br>*Wallemia*<br>*unclassified_p__Chytridiomycota*<br>*Mortierella*
GX & JS & SD only (12)	*Diplodia*<br>*Infundichalara*<br>*unclassified_o__Helotiales*<br>*Issatchenkia*<br>*Trichothecium*<br>*Fusicolla*<br>*Rosellinia*<br>*Rhodosporidiobolus*<br>*Mrakia*<br>*Solicoccozyma*<br>*Dioszegia*<br>*Cutaneotrichosporon*
GX & JX & SD only (4)	*Phialophora*<br>*Xenoacremonium*<br>*Hypoxylon*<br>*Coniophora*
JS & JX & SD only (7)	*Catenulostroma*<br>*unclassified_f__Didymellaceae*<br>*Diutina*<br>*Clonostachys*<br>*unclassified_f__Meruliaceae*<br>*Peniophora*<br>*Cryptococcus_f__Cryptococcaceae*
GX & JS & JX & SD only (47)	*Cladosporium*<br>*Sphaerulina*<br>*unclassified_o__Capnodiales*<br>*Aureobasidium*<br>*Pyrenochaetopsis*<br>*Didymella*<br>*Epicoccum*<br>*Phoma*<br>*Kalmusia*<br>*Paraphoma*<br>*Phaeosphaeriopsis*<br>*Alternaria*<br>*unclassified_o__Pleosporales*<br>*Exophiala*<br>*Bradymyces*<br>*Trichomerium*

	Aspergillus
	Penicillium
	Talaromyces
	Cyberlindnera
	Kazachstania
	Candida
	unclassified_o__Saccharomycetales
	Diaporthe
	Phomopsis
	unclassified_o__Diaporthales
	Gibellulopsis
	Isaria
	Trichoderma
	Fusarium
	unclassified_o__Hypocreales
	Phaeoacremonium
	Pestalotiopsis
	unclassified_c__Sordariomycetes
	unclassified_p__Ascomycota
	Cystidiodontia
	Schizophyllum
	Sistotrema
	unclassified_f__Peniophoraceae
	Symmetrospora
	Malassezia
	Rhodotorula
	Filobasidium
	Apiotrichum
	Trichosporon
	unclassified_p__Basidiomycota
	unclassified_k__Fungi

Table S3. Bacterial and fungal genera used for functional prediction.

Domain	Genus
Bacteria	*Lactobacillus*
	Erwinia
	Brevundimonas
	Pseudomonas
	Enterobacter
	Klebsiella
	Bacillus
Fungi	*Cladosporium*
	Pestalotiopsis
	Trichoderma
	Fusarium
	Alternaria
	Penicillium
	Didymella
	Aspergillus
	Apiotrichum
	Filobasidium

Note: Bacterial and fungal genera ranked among the top 50 in relative abundance in *Pinus massoniana* seeds were compared with taxa reported from adult *P. massoniana* tissues in previous studies, and the shared genera were selected for functional prediction.
